# The Cyclically Seasonal *Drosophila subobscura* Inversion O_7_ Originated From Fragile Genomic Sites and Relocated Immunity and Metabolic Genes

**DOI:** 10.3389/fgene.2020.565836

**Published:** 2020-10-09

**Authors:** Charikleia Karageorgiou, Rosa Tarrío, Francisco Rodríguez-Trelles

**Affiliations:** Grup de Genòmica, Bioinformàtica i Biologia Evolutiva (GGBE), Departament de Genètica i de Microbiologia, Universitat Autonòma de Barcelona, Barcelona, Spain

**Keywords:** non-B DNA, genome fragility, *foxo* (forkhead box subgroup O), *Akt1* (serine/threonine–protein kinase B), *Attacin* antibacterial genes, immunometabolism, thermal adaptation, seasonal selection

## Abstract

Chromosome inversions are important contributors to standing genetic variation in *Drosophila subobscura*. Presently, the species is experiencing a rapid replacement of high-latitude by low-latitude inversions associated with global warming. Yet not all low-latitude inversions are correlated with the ongoing warming trend. This is particularly unexpected in the case of O_7_ because it shows a regular seasonal cycle that peaks in summer and rose with a heatwave. The inconsistent behavior of O_7_ across components of the ambient temperature suggests that is causally more complex than simply due to temperature alone. In order to understand the dynamics of O_7_, high-quality genomic data are needed to determine both the breakpoints and the genetic content. To fill this gap, here we generated a PacBio long read-based chromosome-scale genome assembly, from a highly homozygous line made isogenic for an O_3__+__4__+__7_ chromosome. Then we isolated the complete continuous sequence of O_7_ by conserved synteny analysis with the available reference genome. Main findings include the following: (i) the assembled O_7_ inversion stretches 9.936 Mb, containing > 1,000 annotated genes; (ii) O_7_ had a complex origin, involving multiple breaks associated with non-B DNA-forming motifs, formation of a microinversion, and ectopic repair in *trans* with the two homologous chromosomes; (iii) the O_7_ breakpoints carry a pre-inversion record of fragility, including a sequence insertion, and transposition with later inverted duplication of an *Attacin* immunity gene; and (iv) the O_7_ inversion relocated the major insulin signaling *forkhead box subgroup O* (*foxo*) gene in tight linkage with its antagonistic regulatory partner *serine/threonine–protein kinase B* (*Akt1*) and disrupted concerted evolution of the two inverted *Attacin* duplicates, reattaching them to dFOXO metabolic enhancers. Our findings suggest that O_7_ exerts antagonistic pleiotropic effects on reproduction and immunity, setting a framework to understand its relationship with climate change. Furthermore, they are relevant for fragility in genome rearrangement evolution and for current views on the contribution of breakage versus repair in shaping inversion-breakpoint junctions.

## Introduction

Chromosome inversions are arguably the genetic traits with the earliest and richest record of associations with climate (Hoffmann and Rieseberg, 2008). Research into evolutionary responses to contemporary global warming (Hughes, 2000; Parmesan, 2006) is therefore faced with the challenge of understanding how inversions originate and spread in populations (Kirkpatrick, 2010), while trying to determine their roles in climatic adaptation (Gienapp et al., 2008; Messer et al., 2016).

Chromosome inversions are ubiquitous chromosomal mutations consisting in the reversal of the orientation of a chromosome segment. They originate through either of two major mechanisms, each with its associated distinctive footprints. The first mechanism is intrachromatidal non-allelic homologous recombination (NAHR) between inversely oriented repeats. This mechanism generates inversions with duplications at their ends in both the inverted and uninverted states (Cáceres et al., 1999). The second mechanism is chromosomal breakage and ectopic repair via non-homologous end joining (NHEJ). This mechanism either does not generate duplications or generates them but at the ends of the inverted state only. These two types of NHEJ footprints have been explained in terms of differences in the mode of breakage. Two modes of breakage have been advanced: “cut-and-paste” via clean double-strand breaks (DSBs) that generate blunt ends and staggered. NHEJ inversions without duplications at their ends would originate via cut-and-paste (Wesley and Eanes, 1994), whereas those with inverted duplications at their ends would originate via staggered breaks in one or the two breakpoints. Two staggering models for the origin of the inverted duplications have been proposed (Kehrer-Sawatzki et al., 2005; Matzkin et al., 2005; Ranz et al., 2007): according to the isochromatid model, the duplications would be the filled-in single-stranded overhangs that would result from paired single strand breaks (SSBs) located staggered with each other on opposite strands of the same chromatid (Kehrer-Sawatzki et al., 2005), whereas according to the chromatid model, the duplications would result from unequal exchange between paired sister chromatids, each with one of two paired staggered DSBs at each breakpoint (Matzkin et al., 2005). Note that here the terms *isochromatid* and *chromatid* have switched meanings relative to how they are used in cytogenetics (Savage, 1976). The two staggering models are chromatid models because they assume that inversions originate from either single chromatids during premeiotic mitosis (isochromatid), or paired sister chromatids from the same chromosome during meiotic prophase (chromatid) (Ranz et al., 2007). The models cannot be distinguished based on the pattern of inverted duplications. Yet the chromatid model has been favored over the isochromatid model, because of the length of DNA that would need to be unwound by enzymatic activity in the latter model (Ranz et al., 2007). The chromatid model is also not without potential caveats because NHEJ was found to be suppressed during the meiotic prophase in *Drosophila* (Joyce et al., 2012; Hughes et al., 2018). The prevalence and distribution of the NAHR and NEHJ mechanisms of inversion formation within and across lineages are currently under debate (Ranz et al., 2007; Delprat et al., 2019). The NEHJ mechanism rests upon the occurrence of two or more DSBs. But the source of the DSBs (whether environmental, such as ionizing radiation, or spontaneous, such as non-B DNA-associated sequence instability, where non-B DNA denotes any DNA conformation that is not in the canonical right-handed B form; Lobachev et al., 2007; Zhao et al., 2010; Farré et al., 2015), the relative contributions of breakage versus repair to shaping breakpoint junctions (Ranz et al., 2007; Kramara et al., 2018; Scully et al., 2019), and the relative frequency with which the joined broken ends are from the same chromatid (isochromatid model) versus two distinct sisters (chromatid model) (Ranz et al., 2007) or even, as has been more recently suggested by Orengo et al. (2019), non-sister chromatids (chromosome model) are additional open questions.

Inversions can have direct or/and indirect functional effects (Kirkpatrick, 2010). Direct effects are those ascribable to the mutation *per se*, as it altered the structure or expression of functional sequences at the breakpoints, or the functional neighborhood of genes in the cell nucleus (McBroome et al., 2020). Indirect effects emanate from their associated recombination–suppression effects when in heterozygous condition, whereby they can bind together into close linkage association particular combinations of alleles at genetically distant loci. The evolutionary significance of polymorphic inversions is often thought to chiefly stem from their indirect effects (Dobzhansky, 1947; Wasserman, 1968; Kirkpatrick and Barton, 2006). Although data have been lacking on the relative importance of the two types of effects, there has been renewed interest in using genomics to determine mechanisms for the spread, establishment, and maintenance or fixation of inversions (Corbett-Detig and Hartl, 2012; Corbett-Detig, 2016; Fuller et al., 2016, 2017, 2019; Cheng et al., 2018; Said et al., 2018; Lowry et al., 2019). Because they usually involve many genes, chromosome inversions have enhanced potential for affecting multiple traits, which should expand the opportunities for their maintenance via balancing selection. The extent to which that is the case and the types and transience of the balancing selection mechanisms involved are only beginning to be elucidated (Kapun and Flatt, 2018; Wellenreuther and Bernatchez, 2018; Faria et al., 2019). Amid these unknowns, the inversion polymorphisms of *Drosophila subobscura* emerged among the first genetic traits identified as involved in a species’ adaptation to contemporary climate warming (Rodríguez-Trelles and Rodríguez, 1998, 2007; Balanyà et al., 2006; Rezende et al., 2010).

*Drosophila subobscura* is a native Palearctic species broadly distributed in Europe and the newly invaded areas of North and South America (reviewed in Krimbas, 1992), where it is found generally associated with woodland habitats. It belongs in the obscura group, within which it clusters with the recently derived small-island endemics *Drosophila guanche* and *Drosophila madeirensis*, forming the subobscura three-species subgroup (Bächli, 2020). *D. subobscura* has one of the smallest and least repetitive *Drosophila* reference genomes obtained thus far, which is distributed among five large telocentric chromosomes (A, J, U, E, and O) and one small dot (Karageorgiou et al., 2019). In stark contrast with its two insular relatives, the species has evolved highly rearranged chromosome sequences, which is due to having experienced accelerated fixation rates of paracentric inversions, especially the A sex chromosome. This situation has been interpreted as indicative of the inversions’ role in binding together adaptive alleles in the face of the species’ intense continent-wide gene flow (Karageorgiou et al., 2019). Presently, *D. subobscura* harbors a rich inversion polymorphism, with its five major chromosomes showing parallel adaptive variation patterns across latitude (Ayala et al., 1989), seasons (Rodríguez-Trelles et al., 1996, 2013), and through a heatwave (Rodríguez-Trelles et al., 2013), while rapidly shifting in close association with the ongoing rise in global temperatures (Rodríguez-Trelles and Rodríguez, 1998, 2010; Balanyà et al., 2006). Laboratory attempts to establish the causal nature of this association have, however, largely been inconclusive (Santos et al., 2005; Fragata et al., 2014). Ultimately, a complete understanding of the role of inversions in adaptation to contemporary climate warming in *D. subobscura* will necessarily include the identities and functional properties of the genome sequences affected by them. Advances along this line include the isolation and characterization of breakpoint sequences for 11 of the more than 65 large cytologically visible inversions known for the species, including *A*_2_ (Puerma et al., 2017), *O*_3_ (Papaceit et al., 2012), *O*_4_ and *O*_8_ (Puerma et al., 2016a), *E*_1_ and *E*_2_ (Puerma et al., 2014), *E*_3_ and *E*_9_ (Orengo et al., 2015), *E*_12_ (Puerma et al., 2016b), and *U*_1_ and *U*_2_ (Karageorgiou et al., 2019). An overall conclusion is that none of these inversion breakpoints disrupted any obvious candidate gene for direct adaptation to temperature, despite the fact that all but the *E*_3_ inversion are supposed to be involved in adaptation to climate (e.g., Menozzi and Krimbas, 1992; Rego et al., 2010; Arenas et al., 2018). Apart from the fact that thermal traits are genetically complex and that many of the genes that impinge on them are still unknown, the above conclusion supports that those inversions’ role in thermal adaptation would be through either position effects, indirect linkage generation effects, or both.

**FIGURE 1:**
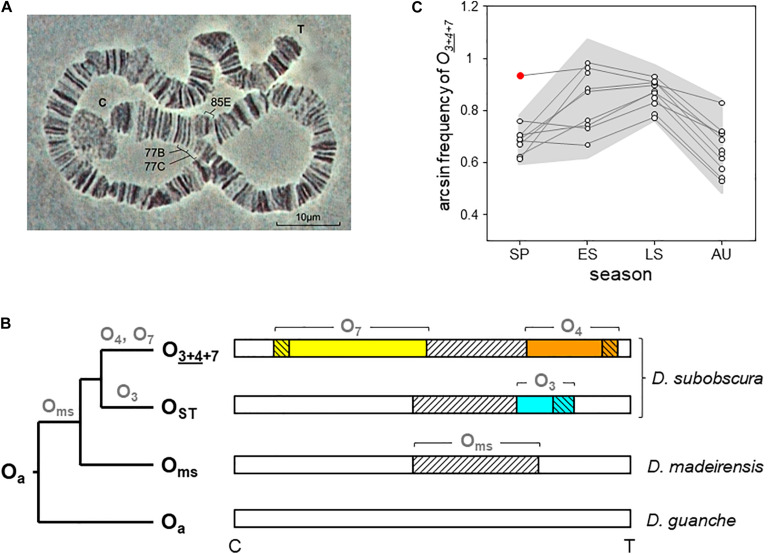
O_7_ inversion and O_3__+__4__+__7_ chromosome arrangement of *D. subobscura*. **(A)** Light micrograph (400 ×) of the O_7_ diagnostic loop from two paired polytene O chromosomes of a O_3__+__4__+__7_/O_3__+__4_ heterokaryotype, with indicated cytological map positions of the two inversion breakpoints (Kunze-Mühl and Müller, 1958; Götz, 1965). C and T denote centromere and telomere, respectively. **(B)** Phylogeny and chromosomal locations of the inversions forming the O_3__+__4__+__7_ arrangement in the subobscura subgroup. Names at the root and tips (bold black) and on branches (bold gray) denote chromosome arrangements and inversions, respectively. The ancestral O arrangement of the subgroup is O_*a*_ (Karageorgiou et al., 2019). The chromosome-central inversion O_*ms*_ (diagonally hatched) is so-called because it became fixed in the last common ancestor of *D. madeirensis* and *D. subobscura* (Karageorgiou et al., 2019). In *D. subobscura*, O_3_ (blue) and O_4_ (orange) are two centromere-distal inversions with overlapping cytological map positions originated independently on separate O_*ms*_ branches. The centromere-proximal inversion O_7_ (yellow) is assumed to have originated along the branch of O_4_. O_*ms*_ became extinct as a single inversion in *D. subobscura*. Note that O_3_ is not in the path from O_*a*_ to O_3__+__4__+__7_, being the inversion that generated the O_ST_ arrangement. **(C)** Five decades of cyclic seasonal change of O_3__+__4__+__7_ at Mount Pedroso, Spain. Consecutive seasonal data (dots) from the same year are connected by lines. The gray background plots the ± 2σ confidence band around the seasonal averages, and the red dot the summer-like value recorded during the spring 2011 heatwave. Included are published data from 1976 to 1981 (Fontdevila et al., 1983), 1988 to 1991 (Rodríguez-Trelles et al., 1996), 2011 to 2012 (Rodríguez-Trelles et al., 2013), and our 2015 unpublished arcsin-transformed records for late summer (0.845) and autumn (0.574). SP, spring; ES and LS, early and late summer; AU, autumn.

As part of a wider effort to develop a high-quality reference genome for *D. subobscura* encompassing the species’ rich chromosomal polymorphisms, here we focus on the O_7_ inversion. The breakpoints of this inversion were located cytologically at subsections 77B/C and 85E on the Kunze–Mühl and Müller *standard* map (Figure 1A; Kunze-Mühl and Müller, 1958; Götz, 1965). O_7_ is among the top 10% known largest *D. subobscura* inversions, stretching most of the centromere-proximal half of the O chromosome (Krimbas, 1992). In nature, it attains significant frequencies only in combination with the non-overlapping centromere-distal complex of two overlapping inversions O_3__+__4_, forming the chromosome arrangement O_3__+__4__+__7_ (Figure 1B). The tight association between O_7_ and O_3__+__4_ is likely maintained by an interaction between selection and the strongly reduced recombination between them (Pegueroles et al., 2010a).

O_7_ could be initially classified as a warm-climate inversion. In the Palearctic, it shows a southern distribution. In northwest Spain, where it has been longitudinally monitored starting in mid-1970s (Fontdevila et al., 1983; Rodríguez-Trelles et al., 1996, 2013), it shows a pronounced regular seasonal cycle (estimated to account for more than 60% of the inversion’s temporal variation; Rodríguez-Trelles et al., 1996) that peaks in summer and drops in winter (Figure 1C). In 2011, it rose to summer-like levels in spring during a heatwave, with the magnitude of the increase closely matching that of the thermal anomaly (Figure 1C; Rodríguez-Trelles et al., 2013). However, (i) the average annual frequency of O_7_ in northwest Spain remains unchanged after decades of sustained climate warming experienced by the region (Rodríguez-Trelles et al., 2013; our unpublished records). (ii) Following the 2011 heatwave, the inversion reached summer-like frequencies in April, but did not continue rising through the ensuing summer (Figure 1C), perhaps hampered by recessive deleterious alleles (Rodríguez-Trelles et al., 2013). (iii) The Palearctic distribution of O_7_ is disjointed between the peninsulas of Iberia and Turkey (Götz, 1967). These are similar latitude areas separated by ∼2,500 km within the continuous species’ range. Assuming that the inversion is molecularly the same in the two areas, this spatial pattern can hardly be explained on the sole basis of a postglacial expansion scenario (Menozzi and Krimbas, 1992), considering how rapidly it spread through the recently invaded areas of the New World (Prevosti et al., 1988). And (iv) in the more studied Iberian Peninsula, the distribution of the inversion has negative or no correlations with the geographical variation in temperature. For example, the average annual frequency of the inversion declines from ∼50% to near-zero values along the > 1,000-km stretching from the northwestern-most to the northeastern-most territories, despite the latter having a warmer climate than the former (de Frutos, 1972; Solé et al., 2002; Rodríguez-Trelles et al., 2013). The same is true for the West Atlantic fringe of the peninsula along which the inversion levels remain basically the same despite the fact that it stretches seven latitudinal degrees of steep thermal gradient (Brehm and Krimbas, 1988; Solé et al., 2002; Rodríguez-Trelles et al., 2013). The inconsistent patterns of O_7_ between components of the ambient temperature suggest that it is influenced by selective factors other than temperature alone.

The O chromosome offers the methodological advantage over the other *D. subobscura* chromosomes that there is an available balancer-strain called *Varicose*/*Bare* (*Va*/*Ba*) (Sperlich et al., 1977). In this study, we first used the *Va*/*Ba* strain to develop an isogenic line with two identical copies of a wild O chromosome carrying the O_3__+__4__+__7_ arrangement. Second, we used PacBio long-read technology to generate a high-quality annotated chromosome-scale genome sequence for the line. Third, we isolated the complete continuous nucleotide sequence of the inversion O_7_ by conserved synteny analysis of the obtained O_3__+__4__+__7_ chromosome with the available O chromosome from the species’ reference genome, which is structurally O_3__+__4_ (Karageorgiou et al., 2019). In addition, we also considered two other published sequences of the O chromosome, including a high-quality long-read–based sequence from *D. subobscura* (Bracewell et al., 2019), and an Illumina-based sequence from *D. guanche* (Puerma et al., 2018). We give an account of O_7_ main features, together with a detailed description of its mechanism of formation. Our findings provide clues to the mixed evidence for this inversion’s role in thermal adaptation.

## Materials and Methods

### Species Karyotype and Inversion Nomenclature

*Drosophila subobscura* shows the ancestral karyotype configuration of the genus *Drosophila*, consisting of five large telocentric rods (Muller elements A-E) and one dot (Muller F) (Powell, 1997). The five rods include the sex chromosome (Muller A) and four autosomes of which the O chromosome (Muller E; homologous to chromosome arm 3R from *D. melanogaster*) is the largest (∼30 Mb), comprising around 25% of the species’ nuclear euchromatic genome (∼125 Mb; Karageorgiou et al., 2019).

An early landmark in the study of chromosomal inversion polymorphisms of *D. subobscura* was the development of structurally homozygous strains, as tools to identify new inversions by the location and shape of the loops formed in inversion heterozygotes (Zollinger, 1950; Maynard-Smith and Maynard-Smith, 1954; Zouros et al., 1974; Loukas et al., 1979). The “Küsnacht” strain, named after the Swiss locality of collection of the flies (Zollinger, 1950), became the first established (Koske and Maynard-Smith, 1954). The chromosomal arrangements of the strain, which happened to be those most common in Central Europe, were subscripted ST (for “standard”) and from them new inversions were designated with numeral subindices following their order of discovery (Kunze-Mühl and Sperlich, 1955). This naming system was not intended to convey polarity of evolutionary change. Accordingly, O_3__+__4__+__7_ is the arrangement that can be interconverted with O_ST_ by the two centromere-distal overlapping inversions O_3_ and O_4_ (denoted by the underline joining the subscripts; Zouros et al., 1974) and the centromere-proximal inversion O_7_. The ancestor-descendant relationships of these inversions are shown in Figure 1B.

### *Drosophila* Lines

O chromosome conserved synteny analysis was based on data from four whole-genome *de novo* assemblies, including three PacBio long-read–based assemblies from *D. subobscura* and one Illumina short-read–based assembly from *D. guanche*. Of the three *D. subobscura* assemblies, one was used as reference for inversion O_7_ and was newly generated in this study. The other two were used as references for the *standard* configuration [note that the distal breakpoint of O_7_ maps within inversion O_*ms*_ (Karageorgiou et al., 2019), whereby is expected to exhibit opposite orientation in *D. subobscura* relative to *D. guanche*; Figure 1B] and were already available (Karageorgiou et al., 2019; Bracewell et al., 2019). Also available was the assembly from *D. guanche* (Puerma et al., 2018), which was used as an outgroup. Henceforth, we will refer to these four assemblies as Ds_7, Ds_ch-cu, Ds_B, and Dg, respectively.

To generate the Ds_7 assembly, we developed a line that is isogenic for an O_3__+__4__+__7_ arrangement from the wild and homokaryotypic and highly homozygous for the ST arrangements of the rest of the chromosomes (i.e., A_ST_, J_ST_, U_ST_, E_ST_, and O_3__+__4__+__7_). The O arrangement was first isolated by crossing wild males to virgin females from the *cherry*-*curled* (*ch-cu*) recessive marker stock; they were then submitted to nine generations of backcrossing with ch-cu females and finally isogenized using the *Va/Ba* balancer stock (Sperlich et al., 1977). The expression of the *Ba* gene is highly variable. Therefore, to prevent potential errors at sorting out phenotypically O_3__+__4__+__7_ homokaryotypes, the *Va/Ba* stock was previously selected for zero macrobristles on the scutum and scutellum. Crossing schemes and the methods for polytene chromosome staining and identification are described elsewhere (Rodríguez-Trelles et al., 1996). The assayed line was stored frozen at −80°C immediately upon obtention. The wild flies used to develop the line were derived from our survey of the natural population of Berbikiz (Spain; Lat.: 43,18949, Long.: –3,09025, Datum: WGS84, elevation: 219 m a.s.l) conducted in July 7, 2012 (Rodríguez-Trelles et al., 2013).

The remaining three assemblies were derived from strains homokaryotypic for all chromosomes. The Ds_ch-cu assembly was generated from the *ch-cu* strain of our laboratory (A_ST_, J_ST_, U_ST_, E_ST_, and O3+4; Karageorgiou et al., 2019) and the Ds_B assembly from an isofemale laboratory stock derived from a natural population from Eugene, Oregon, in 2006 (A_ST_, J_ST_, U_1__+__2_, E_ST,_ and O_3__+__4_; Bracewell et al., 2019). The Dg assembly was generated from an isofemale laboratory stock derived from a natural population from the Canary Islands, Spain, in winter 1999 (Puerma et al., 2018); it shows the chromosome configuration of the last common ancestor of the *subobscura* subgroup except for chromosome E, which carries the arrangement E_*g*__1_ (A_*a*_, J_*a*_, U_1__+__2_, E_*g*__1_, and O_*a*_; Puerma et al., 2018; Karageorgiou et al., 2019; Bracewell et al., 2019).

### High Molecular Weight Genomic DNA Isolation and PacBio Whole-Genome Sequencing

High-quality high-molecular-weight gDNA was obtained from 60 mg of −80°C frozen adult females, using a modified version of the phenol/chloroform method of Chen et al. (2010) that yields ∼25 μg of high-quality DNA per assay, as assessed by NanoDrop ND1000 (NanoDrop Technologies Inc., Wilmington, DE, United States) spectrophotometer and standard agarose gel electrophoresis. The genome of the Ds_7 isogenic line was sequenced to nominal 66-fold genome coverage using PacBio (Pacific Biosciences, Menlo Park, CA, United States) Sequel single-molecule real-time (SMRT) technology from a 20-kb SMRTbell template library, using Polymerase 3.0 chemistry and two SMRT cells. Libraries construction and PacBio sequencing were outsourced to Macrogen (Macrogen Inc., Seoul, South Korea).

### Chromosome-Scale Assembly and Scaffolding

Raw PacBio reads were assembled using the Canu assembler (version 1.8; Koren et al., 2017) on recommended settings for read error correction, trimming and assembly, and genome size set at 150Mb based on previously published flow cytometry data (Karageorgiou et al., 2019). These analyses were performed on a 2.80-GHz 8-CPU Intel Xeon 64-bit 32GB-RAM computer running Ubuntu 18.04 LTS.

Chromosome-scale assembly and scaffolding followed the four steps outlined in Karageorgiou et al. (2019) as well as a fifth step, to improve genome completeness and contiguity, consisting of merging the Ds_7 assembly with a preselected set of segments from the reference Ds_ch-cu assembly using one round of quickmerge (Chakraborty et al., 2016), as follows: first, the CANU contigs that could be certainly anchored, ordered, and oriented on the nuclear chromosomes were aligned against the Ds_ch-cu reference using NUCmer (Kurtz et al., 2004). Second, the segments of Ds_ch-cu not overlapped by the CANU contigs, each extended 10 kb outward from each of its two ends, were extracted. Finally, separately for each chromosome, the extracted Ds_ch-cu segments, together with the CANU contigs set as the backbone, were fed into quickmerge. This approach was found to reduce the chances of misassembly and chimerism, while making it straightforward to trace the non-backbone sequence in the assembly. Dot plots of the merged assembly against the reference Ds_ch-cu assembly were used as a further step of misassembling correction. The obtained Ds_7 assembly was polished with 26 × mean coverage of 150–base-pair (bp) MP Illumina reads from the O_3__+__4__+__7_ isogenic line using two rounds of PILON (version 1.23; Walker et al., 2014).

### Genome Annotation

Gene prediction and annotation of the assembled genome were conducted using the MAKER (version 3.01.02.-beta; Holt and Yandell, 2011; Campbell et al., 2014) annotation pipeline. Repetitive elements were identified using RepeatMasker (version 4.0.6; Smit et al., 2013/2015, at^1^) combined with three repeat libraries, including (i) the *Drosophila* genus–specific repeat library contained in the Repbase database (release 20170127; Bao et al., 2015); (ii) a library of *subobscura* subgroup specific satellites, sat290 and SGC-sat (Karageorgiou et al., 2019); and (iii) a library of *de novo* identified repeats generated using RepeatModeler (version1.0.11) on the assembly masked for the first two libraries. Novel long terminal repeats (LTRs), miniature inverted-repeat transposable elements (MITEs), tandem repeats, and rDNA and tDNA genes were identified using LTRharvest (GenomeTools version1.5.10; Ellinghaus et al., 2008), MITE Tracker (version 2.7.1; Crescente et al., 2018), Tandem Repeat Finder (TRF; version4.09; Benson, 1999), RNAmmer (version 1.2; Lagesen et al., 2007), and tRNAscan-SE (version 2.0; Lowe and Chan, 2016), respectively. All tools were run on default settings, except LTRharvest, for which we set -seed 100, -similar 90.0, and -mintsd 5, following Hill and Betancourt (2018). The quality of the annotation was controlled using the Annotation Edit Distance (AED) metric (Eilbeck et al., 2005). AED values are bounded between 0 and 1. An AED value of 0 indicates perfect agreement of the annotation to aligned evidence, and conversely, a value of 1 indicates no evidence support.

Functional annotation of MAKER-predicted proteins was made by BLASTP (version 2.6.0 +) searches against the *Drosophila* UniProt-SwissProt manually curated datasets (Apweiler et al., 2004). Prediction of protein functional domains was accomplished using InterProScan (version 5.29–68.0; Jones et al., 2014) on the Pfam (Finn et al., 2016), InterPro (Finn et al., 2017), and Gene Ontology (Ashburner et al., 2000; The Gene Ontology Consortium, 2017) domain databases. Genome assembly and annotation completeness were gauged using the Benchmarking Universal Single-Copy Orthologs (BUSCO) tool [BUSCO, version 4 (Seppey et al., 2019)], with the latest update of the dipteran gene set (diptera_odb10), which contains 3,285 highly conserved, single-copy genes expected to be present in any dipteran genome.

### Isolation and Characterization of the O_7_ Breakpoints

Suppose that +A|+B+C|+D and +A|−C−B|+D represent two chromosome arrangements whose gene orders differ only by the orientation of the segment between A and D (with symbols denoting A and D, the segments upstream from the centromere-proximal breakpoint and downstream from the centromere-distal breakpoint, respectively; vertical bars, breakpoint junctions; and plus/minus signs, orientation of the segment relative to the uninverted sequence). We proceeded in two steps. First, we isolated the regions containing the breakpoint junctions by chromosome conserved synteny analysis between the uninverted and inverted states using the Synteny Mapping and Analysis Program (SyMAP, version 4.2.; Soderlund et al., 2011) tool on default options, and NUCmer (see Karageorgiou et al., 2019). The O_7_ breakpoints were identified as the loci of interrupted synteny whose locations and distance from each other agree with the cytogenetic mapping data of the inversion (Karageorgiou et al., 2019). Second, we localized the breakpoint junctions at base-pair resolution and performed comparative analyses of their flanking sequences using the progressive guide tree-based MAFFT algorithm (version 7^2^) with the accuracy-oriented method “L-INS-i” (Katoh et al., 2019). Each of the regions +A|+B and +C|+D from the uninverted state was aligned separately, first with +A|−C and then with −B|+D from the inverted state. From each of the four resulting alignments, we used the regions showing positional homology between the uninverted and inverted states to isolate segments A, B, C, and D, correspondingly. The remaining sequences of the uninverted state were submitted to a second round of comparative analysis among them, and with segments A to D to identify the homologies missed in the first round. As representatives of the uninverted state, we used Ds_ch-cu together with the previously published assemblies Ds_B and Dg, and this last one was set as the outgroup.

### Phylogenetic Inferences

MAFFT-based tree reconstruction of the *Attacin* gene family in *Drosophila* was performed via maximum likelihood. Model selection and tree inference were conducted using IQ-Tree (Kalyaanamoorthy et al., 2017; Nguyen et al., 2015). Tree searches were conducted starting from sets of 100 initial maximum parsimony trees using nearest neighbor interchange with default perturbation strength and a stopping rule settings. Branch support was assessed using the ultrafast bootstrap approximation (UFboot; 1,000 replicates) (Hoang et al., 2018), and two single-branch tests including the Shimodaira–Hasegawa-like approximate likelihood ratio test (SH-aLRT; 1,000 replicates) (Guindon et al., 2010) and the approximate Bayes parametric test (Anisimova et al., 2011).

### Non-B DNA Sequence and Transcription Factor Binding Site Scans

Scans for potential non-B DNA–forming sequences considered the following features: inverted repeats (IRs) (capable of forming hairpin and/or cruciform DNA), direct/tandem repeats (slipped/hairpin structures), mirror repeats (triplexes), alternate purine-pyrimidine tracts (left-handed Z-DNA), G4 motifs (tetraplex and G−quadruplex DNA), and A−phased repeats (static bending). Searches were conducted online using for IRs Palindrome Analyzer (Brázda et al., 2016^3^; accessed January 24, 2020) with repeat length of 6-20 nt, spacer length ≤ 10 nt, and number of mismatches ≤ 1; for tandem repeats Tandem Repeat Finder (TRF version 4.09; Benson, 1999^4^; accessed Jan 24, 2020) in basic mode; and for the remaining features nBMST (Cer et al., 2012^5^; accessed January 24, 2020) with prefixed default settings. The propensity of IRs to adopt non-B conformation was assessed using the difference in free energy between the DNA sequence in the linear and cruciform structures, as implemented in Palindrome Analyser (Brázda et al., 2016).

Transcription start site (TSS) prediction was conducted using the NNPP method (Reese, 2001^6^). Searches for putative binding sites for Relish (*Rel*), the heterodimer Dif/Rel, dFOXO, Dorsal (*dl*), and Serpent (*srp*) transcription factors in the 1-kb upstream region of the *Attacin* predicted TSSs were performed using the FIMO tool (Grant et al., 2011) from the MEME suite (Bailey et al., 2015). For *Rel* and Dif/Rel, and for dFOXO, we used the FootprintDB database (Sebastián and Contreras-Moreira, 2014^7^) *Drosophila melanogaster* Major Position Matrix Motifs (DMMPMM) identified, respectively, by Senger et al. (2004) and Weirauch et al. (2014). For *dl* and *srp*, we used the REDfly database (version 5.5.3; Rivera et al., 2019^8^) improved iDMMPMM motifs developed by Kulakovskiy and Makeev (2009). Searches were performed using a *p* value cutoff of 10^–3^.

## Results

### Chromosome-Scale Assembly and Annotation of Chromosome Arrangement O_3__+__4__+__7_

**FIGURE 2:**
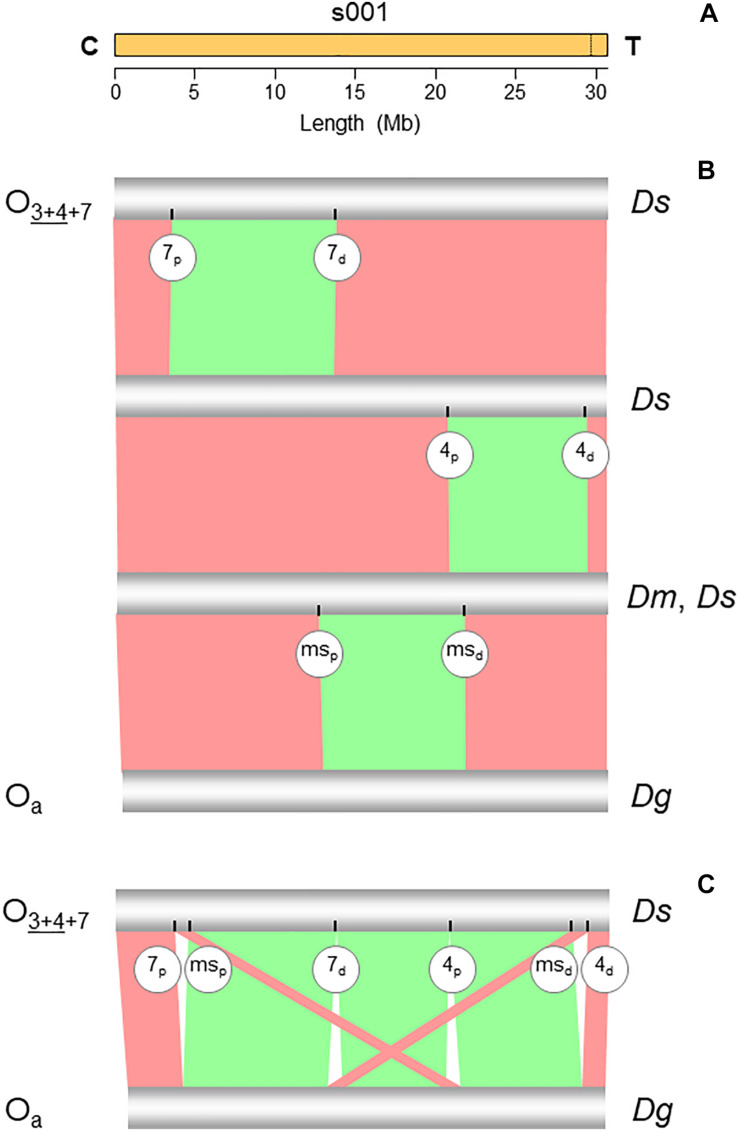
Chromosome conserved synteny analysis of the O_7_ breakpoints. **(A)** The long read-based O_3__+__4__+__7_ chromosome-scale scaffold (s001). The vertical dotted line near the telomere indicates the location of the stitch between the two Canu tigs. **(B)** SyMAP comparative synteny analysis showing the inversions found along the path from the ancestral arrangement of the subobscura subgroup (O_*a*_) to O_3__+__4__+__7_ (see Figure 1B). In addition to O_7_, the two chromosomes also differ by inversions O_4_ and O_*ms*_. **(C)** SyMAP direct comparison of the O_*a*_ and O_3__+__4__+__7_ chromosome arrangements. Bands connecting the chromosomes denote uninverted (pink) and inverted (green) synteny blocks. Labeled ticks on chromosomes indicate proximal (p) and distal (d) inversion breakpoints. The remaining symbols are as in Figure 1.

The PacBio Sequel sequencing of the O_3__+__4__+__7_ isogenic line genome generated 2,457,493 reads, with mean and longest lengths of 11,257 bp and 117,750 bp, respectively. Canu correction and trimming retained a 42-fold genome coverage for the assembly. Of the 385 Canu-generated contigs, the 14 that could be confidently anchored, ordered, and oriented covered the complete reference genome, with an added length of 126.770 Mb and N50 of 10.587 Mb. Quickmerge of those 14 CANU contigs resulted in six chromosome-scale scaffolds, one per each of the major *D. subobscura* chromosomes (Table 1). Of note, chromosome O was built from two contigs only, with the centromere-proximal contig (tig00026085; 29.679 Mb) spanning almost all the chromosome length (96.9%) (Figure 2A). The Ds_7 assembly contained 13,459 MAKER-annotated genes, nearly all with well-supported predictions (AED_50_ = 99.3%). Only 2.6% (87) of the BUSCO genes were missing, indicating that the assembly is almost complete. The O chromosome contained 3,220 (23.9%) of the annotations of the assembly.

**TABLE 1:** Ds_7 assembly summary statistics (Muller elements are given in parenthesis, and lengths are given in megabases of sequence).

Component	Length	Scaffolds	Canu contigs	Largest Canu contig	Gene models
Nuclear genome	126.770	6	14	29.679	13,459
Dot (F)	1.412	1	1	1.412	96
A (A)	22.941	1	2	17.229	2,323
J (D)	25.018	1	3	10.587	2,652
U (B)	26.010	1	3	13.133	2,561
E (C)	20.783	1	3	9.524	2,607
O (E)	30.629	1	2	29.679	3,220

### Identification of Inversion O_7_ Using Chromosome Conserved Synteny Analysis

The structural transition between the O chromosomes of the Ds_7 and Ds_ch-cu assemblies called for one large megabase-sized inversion (Figures 2B,C), whose breakpoints located cytologically precisely as it would be expected if they were from O_7_. Relative to the nearest of the available 140 cytologically mapped markers of the O chromosome (see Karageorgiou et al., 2019), the proximal breakpoint was located 44.5 kb downstream from *Sb* (Dmel\CG4316) and 117.4-kb upstream from microsatellite *dsub02*, and the distal breakpoint 111.8 kb downstream from *rdx* (Dmel\CG12537) and 29.3kb upstream from *Abi* (Dmel\CG9749). *Sb* and *dsub02* have been respectively mapped to subsections 77B (Dolgova, 2013) and 77C (Santos et al., 2010), and *rdx* and *Abi* to subsection 85E (Dolgova, 2013; Pegueroles et al., 2013) of the Kunze-Mühl and Müller (1958) *standard* cytological map. Other than O_7_, no *D. subobscura* inversion maps to those positions.

**FIGURE 3:**
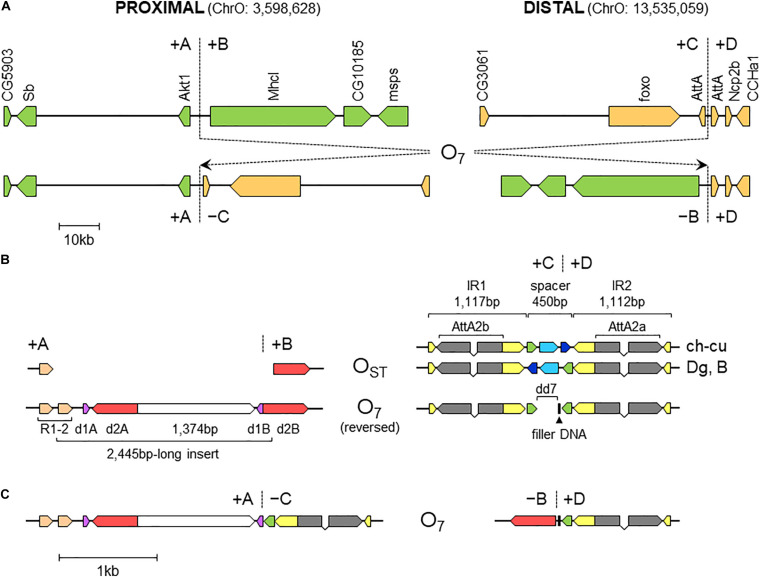
Sequence-annotated breakpoint regions in O_7_ and the standard uninverted state. **(A)** Gene scale (10-kb scale bar) depiction of the proximal and distal breakpoints in the uninverted state (+A|+B and +C|+D, respectively) versus O_7_ (+A|−C and −B|+D). In green are segments A and B, and in sepia C and D. Vertical broken lines indicate break junctions, and arrow boxes the size and direction of the genes labeled vertically using the names of the corresponding *D. melanogaster* orthologs. The coordinates of O_7_ in the assembled chromosome are given in parentheses. **(B)** Zoom-in (1-kb scale bar) on the regions immediately flanking the break junctions, with O_7_ oriented backward (i.e., +B+C, instead of −C−B) to better track the differences with the uninverted state. Arrow boxes indicate the size and direction of the sequence elements discussed in the text. Gray boxes (exons) linked by polygonal lines (introns) represent the two AttA2 paralogs oriented in the direction of transcription. In the distal breakpoint, the two alternative haplotypes of the uninverted states, namely, that from ch-cu and that from Dg and B, are shown. Note the reversal of the spacer in ch-cu versus Dg and B, and the mirror halves flanking dd7 in O_7_. **(C)** O7 represented as in **(B)**, but in its actual orientation (i.e., -C-B).

Comparative analysis of the genes annotated in the regions immediately flanking the breakpoints in Ds_7, Ds_ch-cu and Ds_B with those in the outgroup Dg (Figure 3A) corroborated that Ds_ch-cu and Ds_B carried the uninverted state, whereas Ds_7 carried the inverted state. The assembled O_7_ has a size of 9,936,431 bp, totaling 32.4% of the chromosome (30,629,152 bp). It has a GC content (43.8%) below that of the O chromosome (44.9%) since it is located in the chromosome centromere-proximal half, which is relatively AT-rich (Karageorgiou et al., 2019). O_7_ was predicted to have 1,028 protein-coding genes, or 31.9% of the gene models of the O chromosome, in close agreement with its percent of chromosome length.

### Nature and Properties of the DNA Sequences Surrounding O_7_ Breakpoint Junctions

#### Proximal Breakpoint of O_7_

The alignments used for isolation of the breakpoint junctions and their corresponding flanking regions A, B, C, and D are shown in Supplementary Figures 1, 2. Figure 3B provides a schematic representation of the +A|+B region based on the alignment of Supplementary Figure 3. In the case of O_7_, the region was reconstructed using the reverse complement of segment -B. The breakpoint junction is located within a 2,445-bp-long sequence stretch present only in the inverted state. The site of the insertion is flanked by multiple indels, which suggests that the insertion occurred in a region of prior sequence instability. Of the insertion length, 2,317 bp are on the + A segment and 128 bp on the + B segment. The insertion begins with a 153-bp-long direct repetition (R1-2) of the upstream flank. Proceeding downstream from this repeat, there are two inverted duplications named d1 and d2, each with copies A and B, with d1 shorter (59 bp long each of d1A and d1B) than d2 (534 and 540 bp for copies d2A and d2B, respectively). The two A copies (i.e., d1A and d2A) are separated from the two B copies (i.e., d1B and d2B) by an intervening sequence of 1,374 bp. The junction between + A and + B is precisely located between d1B and d2B. d2B extends 409 bp downward from the downstream end of the insertion into the region of resumed homology between O_7_ and the uninverted state, indicating the orientation of the parent copy.

**FIGURE 4:**
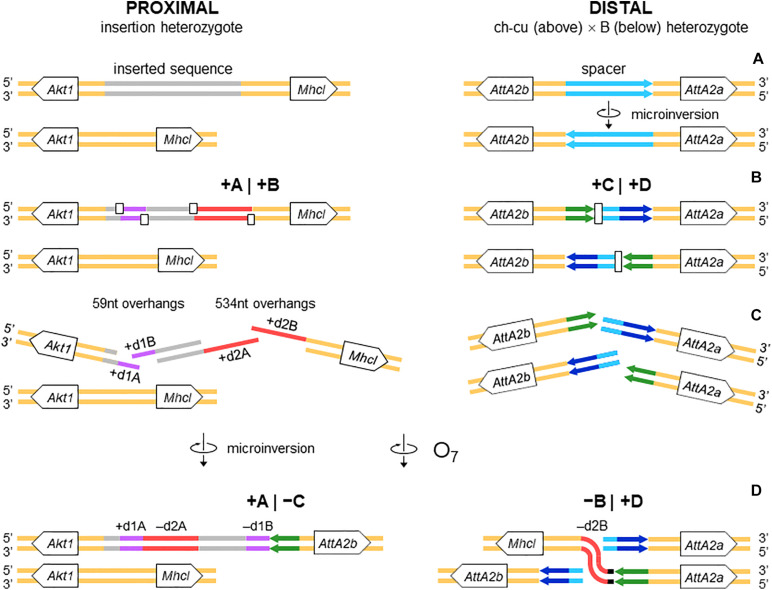
Isochromatid-chromosome mixed staggered model of O_7_ formation. **(A)** Start from an individual heterozygous (or homozygous) for the insertion at the proximal breakpoint, and heterozygous ch-cu–type (above)/B-type (below) for the orientation of the spacer at the distal breakpoint. **(B)** Occurrence of two pairs of isochromatidal staggered SSBs at the proximal breakpoint (open squares), and two staggered chromosomal blunt-ended DSBs at the distal breakpoint (open rectangles), with demarcation of breakpoints flanking segments +A|+B and +C|+D, respectively. **(C)** Emergence of broken ends with single stranded overhangs (+ d1A, + d1B and + d2A, + d2B) at the proximal breakpoint. **(D)** Microinversion formation with fill-in of the overhangs, resulting in the terminal inverted duplications + d1A and -d1B, and the reversed orientation of segment -d2A. And O_7_ formation by reversal of the +B+C segment with fill-in of the -d2B overhang, and distal breakpoint repair via rejoining in *trans* with the homologous chromosome.

The above pattern of sequence copy number, order, and orientation most parsimoniously indicates that the proximal breakpoint of O_7_ was formed on an insertion region that experienced two pairs of staggered SSBs, which resulted in two DSBs (Figure 4; but see section “DISCUSSION” for alternative models). The upstream-most DSB generated the proximal breakpoint of a 2,026-bp-long microinversion and the downstream-most DSB generated a junction flanked upstream by the distal end of the microinversion and downstream by the proximal end of O_7_. Accordingly, duplications d1 and d2 would, respectively, represent the filled-in staggered SSB-induced terminal single-stranded overhangs of the microinversion and inversion O_7_. Figure 3C shows O_7_’s segments A and B such as they are found in the inversion. That d2A and d2B show direct instead of reverse relative orientation as it would be expected if paired–staggered SSBs generate inversions with inverted repeated ends (Ranz et al., 2007) would be explained by the reversal in the orientation of d2A as a result of the microinversion.

Relative to the predicted nearest gene TSSs, the events took place in an intergenic region. Specifically, the upstream-most SSB occurred 1,364 bp downstream from *Akt1* (CG4006; *serine/threonine–protein kinase B*) gene, and the downstream-most one 2,047 nt upstream from *Mhcl* (CG31045; *myosin heavy chain-like*) gene (Figure 3A). From our repeat annotation pipeline, the region around the breakages is a composite of repetitive sequences [16 in total, ranging in length from 21 bp of a (TTG)_*n*_ simple-repeat to 532 bp of satellite rnd-4_family-179], interspersed with traces of transposable elements [84 bp from an LTR and 72 bp from a long interspersed nuclear element (LINE)]. Overall, no evidence of open reading frames and/or specific motifs could be found pointing to the observed breakages as directly caused by the insertion/excision of other sequences.

The role of non-B DNA as source of DSBs is well-established. Generally, DSBs are expected to colocalize with their causal non-B DNA motifs (e.g., Kolb et al., 2009; Lu et al., 2015). We used this prediction to investigate whether the local DNA conformational environment of the ancestral sequence could have acted as trigger or mediator of the complex rearrangement of the proximal breakpoint region. We proceeded in two steps: first, we reconstructed the region of the rearrangement before the breakages. It should be recalled that most of the rearranged sequence is embedded in an insertion that is absent in the ancestral non-rearranged state. Therefore, we reconstructed the prebreakages state by undoing the hypothetical rearrangement steps that generated the present sequence state. Specifically, we reversed the orientation of the microinversion (Supplementary Figure 4) and deleted one copy of each DSB-induced duplication (Supplementary Figure 5). The resulting sequence had the form: + d1, (+ 1,374 bp), |, + d2 (Figure 4B). Which copy of each of the two duplications to eliminate was inconsequential, because they are nearly identical to each other in the two cases (98.3% and 95.6%, for the identities between copies A and B of dup1 and dup2, respectively). Furthermore, the observed high level of identity (97.3%) between d2 and its homologous region in Ds_ch-cu and Ds_B suggested that the rearrangement is recent enough to allow assuming that the original conformational sequence features that could have mediated it are still observable. After establishing the prebreakage sequence, we next looked for sequences with the potential to form non-B DNA structures along a 10-kb window centered on it.

**FIGURE 5:**
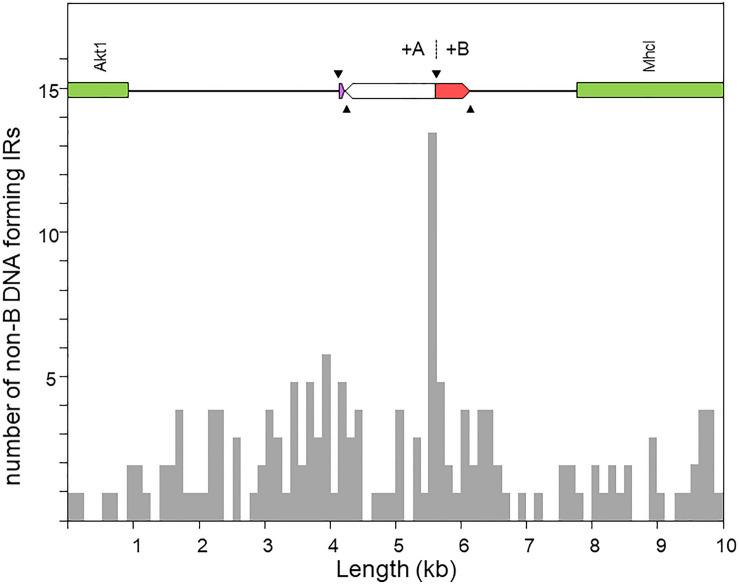
Distribution of inverted repeats (IRs) with potential to adopt non-B DNA hairpin/cruciform structures across a 10-kb window centered at the region of the O_7_ proximal breakpoint, as obtained using Palindrome Analyzer (Brázda et al., 2016). The region of interest is shown at scale above the plot. The purple and red boxes represent respectively the parental copies of d1 and d2 prior to their duplication as a result of the two pairs of staggered SSBs (black arrowheads). The highest concentration of IRs occurs around the junction of the O_7_ breakpoint (+A|+B).

Figure 5 shows the distribution of the number of IRs capable of forming hairpin and cruciform structures along the target sequence. The highest density occurs immediately around the junction between the microinversion and inversion O_7_. In particular, the breakpoint is located within a ∼150-nt-long stretch of AT-rich sequence [simple repeat (ATTT)n, from our genome annotation pipeline] containing 15 IRs, of which one located 68 nt downstream the breakpoint junction ranked in the top 5% with highest likelihood of intrastrand annealing to form a hairpin (AATTTT AAAATT; ΔG_S_ – ΔG_*L*_ = 2.64). In addition, embedded in the IR cluster, there is one tandem repeat of 8.7 copies of the consensus heptanucleotide AATAAAT, and one mirror repeat of two 11 nt-long repeats separated by a 30-nt spacer, indicating that the proximal breakpoint of O_7_ occurred on an unstable sequence with potential for adopting multiple alternative non-B DNA conformations.

#### Distal Breakpoint of Inversion O_7_

Figure 3B provides a schematic representation of the +C|+D region based on the alignment of Supplementary Figure 6. In the case of O_7_, the region was reconstructed using the reverse complement of segment -C. From up to downstream, the breakpoint junction is located within a 450-aligned-sites-long gap-rich spacer region, spanning between two highly identical long IRs, IR1 and IR2, of 1,117 and 1,112 sites of alignment length, respectively. There is no evidence of duplicated sequence in Ds_7 relative to the other assemblies, indicating that the DSB either was a clean cut or did not involve significantly staggered SSBs. On the other hand, the spacer of Ds_7 was the shortest (250 nt) of all four lines (407, 317, and 343 nt for Ds_ch-cu, Ds_B, and Dg, respectively) because of a single deletion located precisely at the center of the region (hereon called dd7, for distal deletion of O_7_). A closer look at the pattern of pairwise sequence similarities along the spacer revealed two findings: (i) dd7 split the Ds_7 spacer in two mirror halves. For the upstream half, Ds_7 is almost identical (96.8%) to Ds_ch-cu while bearing no detectable homology to Ds_B, whereas for the downstream half, Ds_7 is almost identical (97,6%) to Ds_B while bearing no detectable homology to Ds_ch-cu; and (ii) the spacer of Ds_ch-cu is almost identical (95.4%; excluding indels) to that of Ds_B but in reversed orientation. The reversal occurred in Ds_ch-cu, because in Ds_B the spacer is oriented as in the outgroup Dg.

Altogether, the above observations can be understood as follows (Figure 4). Prior to the origination of the distal breakpoint of O_7_, a carrier of an uninverted chromosome of B-type experienced a reversal of the spacer region between the IRs, giving rise to the uninverted chromosome of ch-cu–type. Later on, a homokaryotype for the uninverted chromosome that was heterozygous for the microinversion of the spacer underwent at least two DSBs, one in each of two homologous non-sister chromatids, such that the DSB in the ch-cu–type chromatid occurred immediately before the first site of the dd7 and that in the B-type chromatid immediately after the last site of the dd7. Finally, the reversed + B end generated by the proximal staggered DSB in the ch-cu–type chromatid illegitimately joined with the + D end generated by the distal DSB in its homologous non-sister B-type chromatid, which resulted in a recombinant chromosome carrying the inversion O_7_ with the exact observed dd7 deletion.

Like the proximal breakpoint, the distal breakpoint occurred in an intergenic region yet at comparatively much shorter distance (∼390 bp) to the nearest genes. Specifically, the breakage separated two copies of an *Attacin* gene (CG10146; *AttA*) located opposite to each other on each of the two arms of the long IR. Our repeat annotation pipeline did not identify repetitive sequences in the vicinity of the distal breakpoint in Ds_ch-cu or Ds_B.

We searched the region of the spacer for potential non-B DNA–forming sequences in the vicinity of the breakpoint junctions in Ds_ch-cu and Ds_B. In both cases, we found that the IR with the highest propensity to form a hairpin was a perfect 14-bp-long palindromic sequence located next to the breakpoint junctions (ATGAACT AGTTCAT; ΔG_S_ – ΔG_*L*_ = = 2.05; located 13 and 2 bp upstream and downstream the junction in Ds_ch-cu and Ds_B, respectively). Apart from IRs, we did not detect additional potential non-B DNA sequences around the distal breakpoint.

All nucleotides in the +A|−C region of Ds_7 could be unambiguously ascribed to segment A or C. However, in the −B|+D region −B and +D are separated by 21 extra inserted nucleotides (i.e., GAGCACTCTCCACAGCAAAGT). We decided to ascribe this sequence to the distal breakpoint junction, because it contains an 8-bp substring (underlined) that resembles the beginning of the +D end (CATCAAAG), and hence it likely represents filler DNA generated by a microhomology-templated repair mechanism.

### Pre-inversion Record of Rearrangement of O_7_ Breakpoints

**FIGURE 6:**
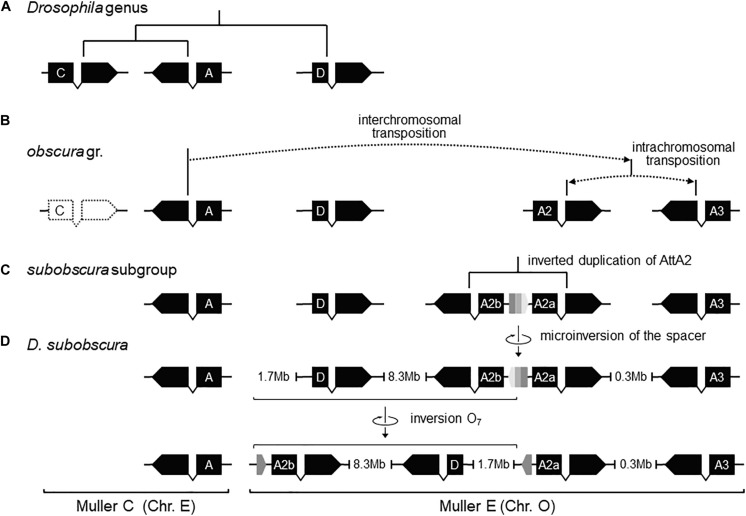
Pre-O_7_ history of instability of the distal breakpoint. **(A)** In the most recent common ancestor of the *Drosophila* genus, *AttD* was the only *Attacin* gene present in Muller element E. **(B)** Later, the ancestor of the obscura group lost *AttC*, and underwent DNA-based interchromosomal transposition of *AttA* (or a close paralog; see Supplementary Figure S7) from Muller C to Muller E, followed by DNA-based intrachromosomal transposition within Muller E, giving rise to *AttA2* and *AttA3* (whether simultaneously or sequentially and, if the latter, which was first is unknown). **(C)** Before the split of the subobscura subgroup, *AttA2* was duplicated, giving rise to the inverted duplicates *AttA2a* (parent copy) and *AttA2b* (daughter) separated by a short central spacer. **(D)** In *D. subobscura*, the central spacer underwent a reversal, generating a microinversion polymorphism with segregating states B-type (ancestral) and ch-cu–type (derived). Genes are represented as solid black boxes (exons) linked by polygonal lines (introns), and oriented in the direction of transcription. The central spacer is represented as a box colored in three shades of gray pointing in the direction of its orientation.

Previously, it was shown that the proximal breakage of O_7_ was preceded by an insertion. Likewise, the region of the distal breakage had a pre-inversion history of rearrangement, which run closely associated with a highly dynamic evolution of the *Attacin* immunity gene family in the *obscura* species group. This conclusion is based on phylogenetic analysis of the *Attacin* family in *Drosophila* (Supplementary Figure 7) using synteny to distinguish orthologous from paralogous copies (Supplementary Table 1). The results are summarized in Figures 6A–D. The most recent common ancestor of the *Drosophila* genus (Figure 6A) carried three copies of the gene with relationships [(A,C),D], of which the more distant D was located in Muller element E, and the closer to each other A and C in Muller element C. After it split from the *melanogaster* group (Figure 6B), the branch leading to the *obscura* group lost copy C and underwent an interchromosomal transposition of copy A from Muller element C to E. The daughter copy then underwent another, in this case intrachromosomal, transposition, which originated two new *Attacin* copies that we called *AttA2* and *AttA3*, with *AttA2* located between *foxo* and *Npc2b*, and *AttA3* located ∼300 kb downstream from *AttA2*, between *Cul5* and *Sirt7*. The two transpositions were genome-based duplications rather than retroposition events, because the new copies conserved the intron position of their parental gene. Before the split of the *subobscura* subgroup (Figure 6C), copy *AttA2* underwent an inverted duplication that generated the two closely spaced copies *AttA2b* and *AttA2a* in head-to-head orientation, and transcribed in opposite directions. In *D. subobscura* (Figure 6D), the spacer between the IRs experienced a reversal of orientation generating the microinversion polymorphism of the distal breakpoint. Subsequently, a heterozygote for the microinversion underwent distal DSBs that allowed the formation of the recombinant O_7_ inversion via ectopic repair of non-sister chromatids.

### Potentially Functional Effects of the O_7_ Mutation

**FIGURE 7:**
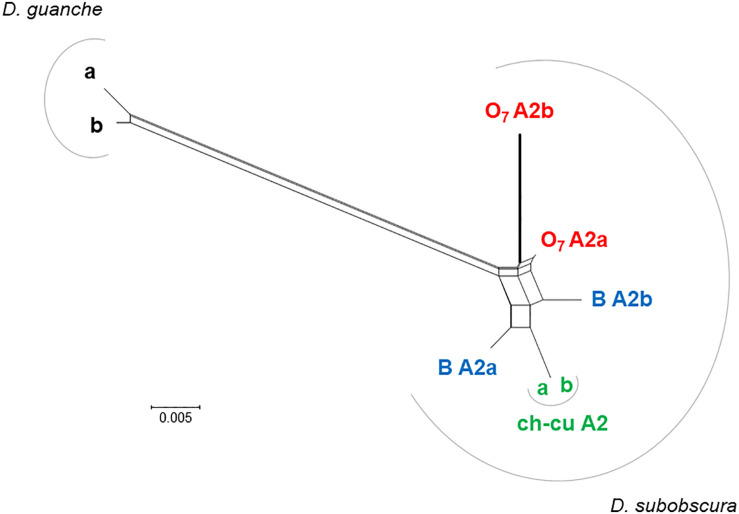
Phylogenetic network of *AttA2* orthologous and paralogous sequences from the inverted (O_7_) and uninverted (B and ch-cu) states in *D. guanche* and *D. subobscura*. The duplication is older than the species, but the paralogs cluster within species owing to concerted evolution. Depicted thicker is the branch leading to the copy relocated by O_7_ (i.e., *AttA2b*), which is longer than that leading to the copy that remained in place (*AttA2a*) due to an acceleration of the synonymous substitution rate, likely as a result of having escaped concerted evolution. The split network was constructed using the NeighborNet method as implemented in SPLITSTREE version 4.14.5 (Huson and Bryant, 2006), on the JC69 + I (% of invariable sites 81.6) best-fit model distances obtained using the DIVEIN web server (https://indra.mullins.microbiol.washington.edu/DIVEIN/) (Deng et al., 2010). Sets of parallel edges represent conflicting topological signals.

The distal break of O_7_ disrupted concerted evolution between two subobscura subgroup-specific *AttA2* duplicates. This conclusion is based on the previous section’s results, together with the phylonetwork of coding sequences shown in Figure 7. Accordingly, right after the duplication of *AttA2*, the two paralogs began to evolve in concert, converting each other to generate their present characteristic phylogenetic pattern of greater resemblance between paralogs from the same species (i.e., *D. guanche* and *D. subobscura*) than between orthologs from different species (e.g., Puig-Giribets et al., 2019). At one end of the resemblance, it is the ch-cu strain, whose two *AttA2* copies are identical to each other, and at the other end O_7_, where the copy relocated by the inversion evolved significantly faster than the one that remained in place, owing exclusively to an acceleration of the synonymous substitution rate [*P* < 0.05; Tajima’s relative rate test (Tajima, 1993) using either of the remaining six sequences as outgroup], as the two copies are identical at the amino acid level. The acceleration took place in the direction of a slight decrease in codon bias in the relocated copy (Nc = 51.2 vs. 50.7, for the comparison *AttA2b* vs. *AttA2a*, respectively; where Nc is the improved effective number of codons index; Sun et al., 2013). The increased synonymous rate can be understood, in part because the inversion released the two *Attacin* copies from evolving in concert; and in part assuming that the expression of the paralogs shifted as a result of changes in regulatory environment associated with their relocation.

**FIGURE 8:**
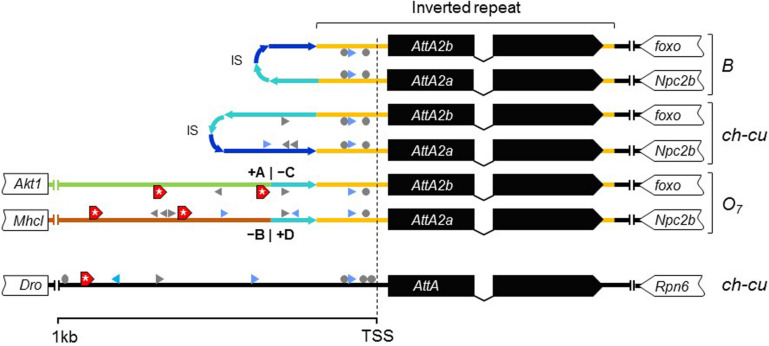
New dFOXO binding sites. Schematic representation of up to 1-kb sequence up and downstream predicted TSSs of *AttA* and the inverted duplicates of *AttA2* (represented as in Figure 6) in O_7_ and the uninverted (B and ch-cu) states, including also the nearest flanking genes. Colored lines connecting genes designate the following: orange, region of the inverted repeats; dark and light blue, first and second halves of the spacer of the inverted repeats, respectively, oriented as the arrowheads; green and brown, the novel sequences to which the *AttA2* copies became reattached by O_7_, with corresponding breakpoints (+A|−C and −B|+D) indicated. The inverted repeats of B and ch-cu are folded over each other. Putative TFBs are symbolized: gray arrowheads and circles (palindromic sites), for Dorsal, Relish, and Diff/Relish; blue arrowheads for Serpent, and red boxes with an asterisk for dFOXO, respectively. Only *AttA* and the two *Att2A* copies of O_7_ have TFBs for dFOXO.

Considering the short spacing between the two *AttA2* paralogs in the uninverted chromosome (∼390 bp), it appeared likely that the inversion would have detached them from part of their promoters, binding them to new potentially *cis*-acting elements. To assess this possibility, we searched 1 kb upstream of the predicted TSS of each gene for putative transcription factor binding sites (TFBSs) for five transcription factors (TFs), including the nuclear factor κB factors dorsal (dl), dorsal-immunity related factor Dif and Relish (Rel), the GATA factor Serpent (*srp*), and the forkhead factor dFOXO. The first four TFs are under control of the Toll and immune deficiency (IMD) immunity pathways and regulate *Attacin* inducible expression in response to bacterial infection (Senger et al., 2004). dFOXO TF is controlled by the insulin/insulin-like growth factor signaling (IIS) metabolic pathway and regulates constitutive *Attacin* expression in non-infected flies suffering from energy shortage or stress (Becker et al., 2010). The results are shown in Figure 8. The *AttA2* genes had predicted TFBSs for the immunity related factors in both uninverted and inverted chromosome states, but only the *AttA2* genes of the inverted chromosome had TFBSs for the metabolic factor dFOXO. Furthermore, the dFOXO TFBSs were all contributed by the newly attached sequence. The fact that the *AttA2* genes were conserved at the amino acid level in *D. subobscura*, together with the observed qualitative difference in predicted *cis*-acting sequence between uninverted and inverted chromosomes, suggests that the inversion O_7_ brought the *AttA2* genes under the influence of the IIS metabolic pathway.

In addition to the *Attacin* immunity genes, the breakpoint regions include *Akt1* and *foxo*, two interacting core components of the IIS metabolic pathway identified by other studies as candidate for climate adaptation (Fabian et al., 2012; Paaby et al., 2014; Kapun et al., 2016; Durmaz et al., 2019). The roles of these genes and the potential impact of O_7_ on them are dealt with in the *Discussion*.

## Discussion

### Molecular Mechanism of O_7_ Formation

#### O_7_ Is a Complex Multibreak Inversion Formed via Rejoining in *trans* With the Two Homologous Chromosomes

Sequence data on inversion formation in *Drosophila* have been interpreted in terms of two major mechanisms with associated distinctive footprints. The first mechanism is intrachromatidal NAHR between inversely oriented repeats. This mechanism generates inversions with duplications at their ends in both the inverted and uninverted states (Cáceres et al., 1999), which is not the case of O_7_.

**FIGURE 9:**
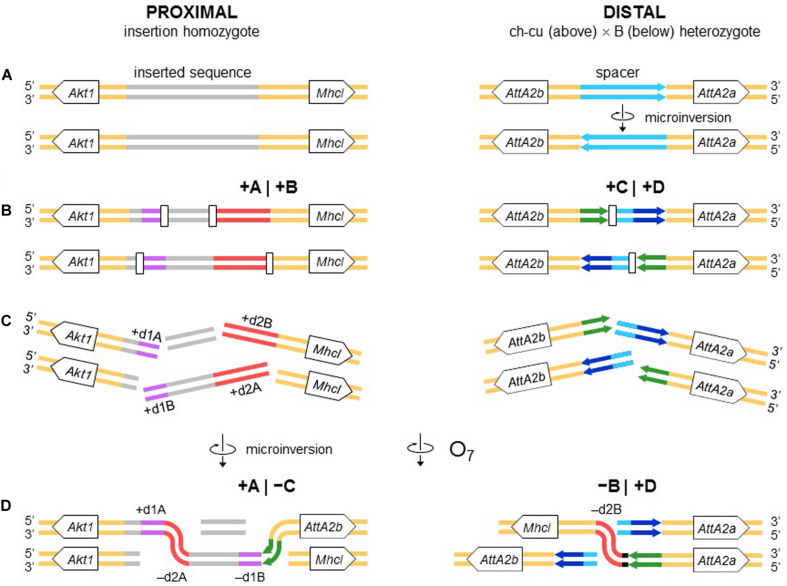
Chromosome model of O_7_ formation. **(A)** Start from an individual homozygous for the insertion at the proximal breakpoint, and as in Figure 4 at the distal breakpoint. **(B)** Occurrence of two pairs of staggered chromosomal blunt-ended DSBs (open rectangles) at the proximal breakpoint, and as in Figure 3 at the distal breakpoint. **(C,D)** Microinversion formation, and formation of O_7_ via rejoining in *trans* with the homologous chromosome as indicated. The model results in an order of the duplications (i.e., + d1A, -d2A, -d1B, -d2B) identical to that resulting from the model of Figure 4.

The second mechanism is chromosomal breakage and ectopic repair via NHEJ. This mechanism either does not generate duplications or generates them but at the ends of the inverted state only. These two types of NHEJ footprints have been explained in terms of two alternative modes of breakage: cut-and-paste via clean DSBs that generate blunt ends and staggered on the same (isochromatidal) or different (chromatidal) sister chromatids (see *Introduction*). In the case of O_7_, it is not a cut-and-paste inversion, but neither is it a typical staggered breaks inversion. Thus, while the inversion proximal breakpoint could be either isochromatidal (Figure 4) or chromatidal (Figure 9), the distal breakpoint has to involve the two homologous chromosomes (Figures 4, 9). This latter pattern could be deduced because of the chanceful circumstance that our two representatives of the uninverted state (i.e., Ds_ch-cu and Ds_B) segregated for the microinversion of the spacer between the IRs flanking the distal breakpoint. Alternatively, the distal breakage could have occurred in a recombinant between chromosome types ch-cu and B. This, however, appears unlikely because crossover within microinversions should be extremely rare (Greig, 2007). Our conclusion agrees with a study of the genealogical relationships between inversions of the E chromosome in *D. subobscura*, which proposed that E_9_ arose in a heterokaryotype E_ST_/E_1__+__2_ to accommodate a conflict between molecular and cytological data (Orengo et al., 2019). This and our results indicate that NHEJ inversions form through mechanisms that can incorporate information from the two homologous chromosomes (chromosome model), in addition to the previously proposed intrasister and intersister chromatidal exchanges.

#### The Breaks of the O_7_ Inversion Were Likely Induced by Non-B DNA Secondary Structures

Inversion O_7_ provides, to our knowledge, the first compelling evidence for a role of non-B DNA in inversion formation in *Drosophila*. Previous studies had reported the presence of AT-rich sequences around the breakpoints of some fixed (Cirera et al., 1995; Richards et al., 2005) and polymorphic (Prazeres da Costa et al., 2009) inversions. In no instance, however, were particular sequences susceptible to adopt secondary structures identified. In the case of O_7_, the proximal break junction occurred just within a palindromic AT-rich repeat capable of adopting hairpin/cruciform, slipped and triplex DNA conformations. Likewise, the distal junctions are located next to perfect 14-bp-long hairpin/cruciform-forming palindromes.

The role of non-B DNA-forming sequences in causing genome instability is well-established (Wang and Vasquez, 2006; Lobachev et al., 2007; Aguilera and Gómez-González, 2008; Zhao et al., 2010). The shift from B to non-B DNA conformation occurs while DNA is in single-stranded form, e.g., behind replication forks, between Okazaki fragments, or in actively transcribed genes (Voineagu et al., 2008). Non B-DNA structures induce DSBs through, e.g., stalling replication and transcription (Mani and Chinnaiyan, 2010; Kaushal and Freudenreich, 2019). There are no specific predictions as to the type, number, and location of the DSBs generated by any given structure in any particular situation. Still, a single structure can induce multiple DSBs across hundreds of base pairs around it (Wang et al., 2006; McKinney et al., 2020), and stalled replication forks can accumulate up to 3 kb of single-stranded DNA (Sogo et al., 2002; Lopes et al., 2006). In the case of O_7_, this length is well over the size of the overhangs that would be generated by an isochromatid model of the proximal breakpoint (58 and 534 nt; see Figure 3 and Supplementary Figure 3).

#### The Inverted Duplications at the O_7_ Breakpoints Could Be Footprints of Repair Instead of Staggered Breakage

**FIGURE 10:**
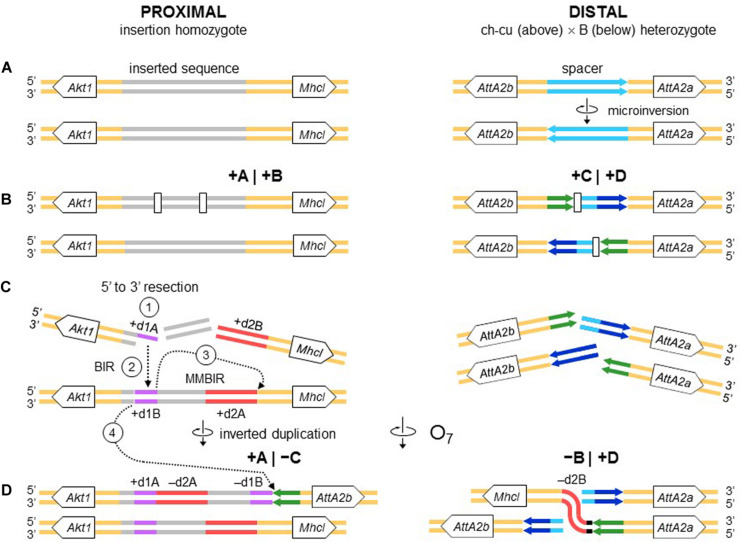
Chromosome and BIR/MMBIR repair model of O_7_ formation. **(A)** Start as in Figure 9. **(B)** Occurrence of one pair of blunt-ended DSBs (open rectangles) at the proximal breakpoint of the ch-cu type chromosome, and as in Figure 4 at the distal breakpoint. **(C,D)** Step 1: 5′ to 3′ resection generating a 3′ single stranded + d1A end. Step 2: beginning of a BIR event via strand invasion into the homologous region of the B-type chromosome. Step 3: switch from BIR to MMBIR, with forward template switching to the distal end of + d2A and backward copying. Step 4: MMEJ to the distal break-end of O_7_ from the original ch-cu–type chromosome. The distal breakpoint repaired as in Figure 9. The model results in an order of the duplications (i.e., + d1A, -d2A, -d1B, -d2B) identical to that resulting from the model of Figures 4, 9.

All the aforementioned inverted duplication-generating NHEJ models are predicated upon the role of DNA breakage (Ranz et al., 2007). However, the inverted duplications at the ends of O_7_ could also be explained as a result exclusively of repair, with no need for invoking staggering of the breaks. DNA repair has emerged as a key factor capable of generating extremely complex breakpoint sequence rearrangements (reviewed in Scully et al., 2019). The spectrum of known error-prone repair mechanisms can be grossly classified as recombination-based, such as microhomology-mediated end-joining (MMEJ), and replication-based, such as break-induced replication (BIR) and microhomology-mediated BIR (MMBIR) (Lee et al., 2007; Zhang et al., 2009; Hastings et al., 2009). Here, the term *microhomology* is used to mean a short tract (∼1 – 25 bp) of chance similarity, rather than common descent. In the case of O_7_, three features suggest that what appear to be footprints of breakage by the staggering models could in fact be footprints of a replication-based mode of repair (reviewed in Kramara et al., 2018; Scully et al., 2019), including (i) presence of non-B DNA-forming sequences just in, or adjacent to breakpoint junctions (see below); (ii) spatial proximity of the breakpoint regions in the nucleus, as evinced by the fact that the genes flanking the junctions are closely related functionally (Farré et al., 2015; but see Sunder and Wilson, 2019); and (iii) multiple breaks concentrated in a short sequence segment. A fourth feature, namely, presence of microhomology at the distal breakpoint junction, would be also consistent with a recombination-based mechanism such as MMEJ. Overall, these features suggest that O_7_ arose as result of a non-B DNA-induced replication impairment, affecting at least its proximal breakpoint. It is known that this type of events can trigger BIR and MMBIR repair (Sakofsky et al., 2015). Of the two pathways, the second pathway has yet to be identified in *Drosophila* (Alexander et al., 2016; Bhandari et al., 2019). A possible scenario is detailed in Figure 10: first, non-B DNA-induced stalling of a replication fork at the proximal breakpoint of a ch-cu–type chromosome led to two DSBs generating three fragments. Second, the centromere-proximal fragment engaged in a BIR event using the homologous region of a B-type chromosome. Third, a second fork stalling triggered a switch from BIR to MMBIR with template switching to a downstream microhomology. Copying backward from the new template resulted in the rearrangement of the proximal breakpoint, including the inverted duplication of the O_7_ end (e.g., Lee et al., 2007; Smith et al., 2007; Carvalho et al., 2015; Tremblay-Belzile et al., 2015). Finally, the event was terminated by an MMEJ to the distal break-end of O_7_ from the original ch-cu-type chromosome (e.g., Scully et al., 2019).

#### The O_7_ Breakpoints Carry a Pre-inversion Record of Fragility

The breakpoint sequences of O_7_ had a record of instability prior to the origin of the inversion, as evinced by the fact that they are located within sequences inserted from elsewhere in the genome. This suggests that the regions that gained those insertions were relatively exposed in the nucleus (reviewed in Farré et al., 2015). In the case of the proximal breakpoint, that could be associated with high levels of transcriptional activity at the broadly expressed *Akt1* gene (Andjelković et al., 1995; Slade and Staveley, 2016).

That the O_7_ junctions arose in fragile regions, beyond the proximate effects of their associated non-B DNA (see above), may be most apparent from the pre-inversion record of recurrent rearrangement of the IR at the distal breakpoint (Figure 6). This record is particularly amenable to reconstruction because the IR largely consists of two copies of the *Attacin A* gene that are highly conserved. It includes at least three rearrangements that occurred in the lineage of *D. subobscura* after its separation from that of the *melanogaster* group (see section “RESULTS”; Figure 6), namely, (i) insertion of *AttA2* between the *foxo* and *Npc2b* genes; (ii) emergence of the IR by inverted duplication of the parental *AttA2* (Figure 6B), which could have occurred through an event of forward template switching and backward copying by the DNA polymerase (Smith et al., 2007; Lee et al., 2007), as discussed above; and (iii) emergence of the ch-cu–type chromosome via inversion of the spacer between the IRs in a B-type chromosome (Figure 6D), which could be explained as an outcome of a stem-loop formation by the IR, followed by resolution of the strand-exchange junctions between the IR arms (see Figure 4 in Leigh Brown and Ish-Horowicz, 1981; Figure 3 in Kolb et al., 2009 and Zhao et al., 2010).

The pre-O_7_ insertion in the proximal breakpoint is specific to *D. subobscura* and is therefore much more recent than that of *AttA2* in the distal breakpoint. Preliminary analyses indicate that it is internally rearranged relative to other paralogous copies, supporting that it carries recombinogenic potential. The origin and evolution of this inserted sequence, as well as its possible implication in the formation of other *D. subobscura* inversions, warrant further investigation (CK, RT, and FR-T; manuscript in preparation).

### O7 Breakpoints Potentially Functional Effects

#### O_7_ Relocated *foxo* in Tight Linkage Association With Its Antagonistic Regulatory Partner of the IIS Metabolic Pathway *Akt1*

O_7_ changed *foxo* from being megabases (∼10 Mb) away from *Akt1* to being tightly linked to it, with only the short *AttA2b* gene sandwiched between them. *Akt1* and *foxo* are functionally conserved genes, which, in *Drosophila*, encode the serine/threonine–protein kinase B AKT/PKB, and the forkhead-box DNA-binding domain-containing TF dFOXO, respectively. The two genes are key antagonistic regulators of the IIS pathway (Teleman, 2010; Slade and Staveley, 2016), a major trigger of shifts in anabolic versus catabolic cellular activity in response to nutritional status (de Jong and Bochdanovits, 2003) and multiple other cues (Regan et al., 2020). In abundant nutrient conditions, AKT/PKB inactivates dFOXO, thus shifting food energy allocation toward reproduction and growth (the IIS pathway). Conversely, scarce nutrient conditions prevent AKT/PKB from inactivating dFOXO, which redirects metabolism toward mobilization of energy stores for somatic maintenance (FOXO pathway). Laboratory research using large effect mutants has shown that the IIS/FOXO pathway is extensively pleiotropic, with major evolutionary conserved effects on fitness-related life-history traits, including growth, size, reproduction, lifespan, and stress resistance (reviewed in Flatt and Partridge, 2018). Research from the field found IIS loci to harbor substantial genetic variation, which frequently exhibits spatiotemporal patterns that look as if they were shaped by selection on the associated IIS traits (Fabian et al., 2012; Paaby et al., 2014; Kapun et al., 2016). In a recent laboratory assay, two *foxo* alleles showing opposite latitudinal clines in *D. melanogaster* were compared on an otherwise homogeneous genetic background. The alleles showed contrasting effects on viability, size-related traits, starvation resistance, and fat content, whose directions were overall consistent with predictions from the clinal variation of the characters (Durmaz et al., 2019).

The O_7_ mutation could have altered *Akt1* and/or *foxo* function via multiple non-mutually exclusive mechanisms, such as mutual regulatory interference, considering that they are antagonistic effectors; relocation to the sides of an immunity gene (i.e., *AttA2b*) expected to be under intense purifying selection on expression (see below); and alteration of the genes’ functional neighborhood at higher-order levels of chromatin organization (Farré et al., 2015; McBroome et al., 2020). It could be argued that the nuclear environment of the genes remained basically unaltered, if the reason why they became involved in the rearrangement was that they already were in close spatial proximity to each other in the nucleus. This, however, did not necessarily have to be the case, considering recent findings in yeast that rejoining of DNA break ends is not determined by the predamage spatial proximity of the DSBs (Sunder and Wilson, 2019). Be that as it may, bearing in mind that the seasonal increase of O_7_ occurs from early spring to midsummer, coinciding with the growth season, it seems more likely that whatever the effect of the inversion mutation on *Akt1* and/or *foxo*, it occurred in the direction of an enhanced basal IIS versus dFOXO activity relative to the O_ST_ ancestral state. This would raise the question of why the O_7_ frequencies decrease (and those of O_ST_ increase) every year from late summer to winter.

#### O_7_ Disrupted the Concerted Evolution of Two *AttA2* Immunity Genes and Reattached Them to Putative dFOXO Metabolic Enhancers

The immune function is highly energy demanding in terms of both maintenance and, especially, rapid deployment upon infection (reviewed in Dolezal et al., 2019). Therefore, within a limited energy budget, a trade-off is expected between reproduction and immunity (Schwenke et al., 2016). The *Drosophila* innate immune response consists of a cellular and a humoral component. The humoral component involves the production of antimicrobial peptides, among which Attacins are active against gram-negative bacteria (Hanson and Lemaitre, 2020). The two main modes of Attacin production, including the induced (by a factor of even > 100) upon infection mode, and the basal in absence-of-infection mode link immunity with the *Akt1*/*foxo* IIS metabolic signaling pathway (Becker et al., 2010; Dolezal et al., 2019). The inducible mode is regulated primarily by the immunodeficiency *Imd* signaling pathway and to a lesser extent by the *Toll* signaling pathway. The two signaling pathways have the same effect of activating dFOXO, thus mobilizing resources toward the production of Attacins (Dionne et al., 2006; Dolezal et al., 2019). The basal mode is regulated directly by dFOXO activity when induced by starvation (Becker et al., 2010; Buchon et al., 2014). Immunity genes, including *Attacins*, are among the known most rapidly evolving genes and have frequently shown evidence of local adaptation in *Drosophila* (Lazzaro and Clark, 2001, 2003).

There would be a number of mechanisms by which the *O*_7_ mutation could have reduced *Attacin* genes’ expression. For example, the breakage of the invertedly transcribed *AttA2* tandem duplicates could have impaired the inducibility of one or the two paralogs, or their separation could have made them lose gene expression coregulation, as might be surmised from the observations that they halted or slowed down evolving in concert, and that *AttA2b* shows decreased codon bias. These mechanisms could have acted synergistically with each other and with those already discussed in connection with *Akt1* and *foxo*. Although this scenario could be partially offset by the increase in basal *AttA2* transcript levels that may be expected from the duplicates having been reattached to dFOXO enhancers (Becker et al., 2010), all in all, the evidence suggests that (i) at its inception, O_7_ caused a rearrangement with partial disruption of a set of functionally related loci with overlapping pleiotropic effects on immunometabolic traits. If, in addition to these direct effects, there concurred indirect effects of linkage between locally, and given the functional relationship, likely epistatically interacting alleles warrant further investigation; and (ii) the resulting haplotype imparted a shifted pattern of resource allocation toward reproduction at a cost to immunity, compared to the O_ST_ ancestor. Such an opposing antagonistic pleiotropy would result in a seasonal frequency cycle qualitatively similar to that shown by the inversions, if reproduction is favored from early spring to midsummer, when O_7_ rises (and O_ST_ wanes), and immunity from late summer to winter, when it wanes (and O_ST_ rises). There is ample evidence that the qualitative and quantitative composition of temperate bacterial communities cycles seasonally (Lazzaro et al., 2015; Shigyo et al., 2019). Recently, a study using *D. melanogaster* from the eastern United States (Behrman et al., 2018) found a seasonal shift in immunocompetence, with the trait value declining every spring to autumn. The shift was interpreted as resulting from relaxed selection for immune response during the warm season, much like what we propose here for the O_7_/O_ST_ inversion polymorphism.

Prior data on temporal genetic variation within and between O inversions point to additional loci that would be consistent with the seasonal cycle of O_7_ being mediated by immunometabolic selection (Rodríguez-Trelles, 2003). The case of the *Mpi* gene encoding the key glycolytic enzyme mannose-6-phosphate isomerase (MPI) is noteworthy. From our assembly, *Mpi* is located 2.15 Mb outward from the distal breakpoint of O_7_, which is within the estimated region of the inversion-associated strong recombination–suppression effect (3.5 Mb; Pegueroles et al., 2010b). The MPI fast/slow electrophoretic polymorphism was found to be only moderately associated with the O_7_/O_ST_ polymorphism. Yet (i) the magnitude of the locus-by-inversion disequilibrium cycled seasonally, and (ii) the cycling occurred because the Fast allele increased in frequency every winter only within the O_7_ chromosomal class, but not within the O_ST_ class (Rodríguez-Trelles, 2003). The behavior of *Mpi* could be in part an outcome of hitch-hiking with other linked loci involved in seasonal adaptation. One such candidate could be the *Na pump*α *subunit* (*Atpα*) gene, located only 0.13 Mb farther away from O_7_ than *Mpi*, and recently found to be under positive selection for defense against plant secondary compounds in *D. subobscura* (Pegueroles et al., 2016). Still, immune elicitation in *Drosophila* relies upon massive upregulation of glycolysis (Dolezal et al., 2019), which should place a strong demand on MPI activity (Shtraizent et al., 2017). In addition to the evidence from *D. subobscura* just discussed, Supplementary Table 2 provides additional loci found to exhibit seasonal variation in a genomic survey from other *Drosophila*, which may be candidates for being involved in the seasonal cycling of O_7_.

## Conclusion and Outlook

Previous work on the spatiotemporal distribution patterns of the inversion polymorphisms of *D. subobscura* indicated that O_7_ is driven by selective factors other than temperature alone. Here, we addressed this issue using a genome-based approach to isolate and characterize the O_7_ breakpoints. Our findings have general implications for current theories on the molecular mechanisms of formation of this common type of structural genomic change. Furthermore, they suggest that O_7_ may have altered fly’s immunometabolism through at least direct effects on core immunity and metabolism genes. This result could help to explain the inversion’s conflicting correlations with the seasonal and decadal climate changes, taking into account recent findings from microbial ecology, which indicate that microbial community responses to short- and long-term climate changes can be largely uncorrelated (Romero-Olivares et al., 2017). Considering its large size, it seems likely that O_7_’s evolution is also shaped by additional direct or/and indirect effects on genes other than those near its breakpoints. Further progress along this line will include development of functional tests of the identified genes on inverted versus uninverted chromosome backgrounds and use of the obtained assembly for building a SNP panel for O chromosome-wide scans of selection. We have incorporated the chromosome-scale sequence of O_3__+__4__+__7_ obtained here into our reference genome browser^9^ to facilitate the further use of this resource.

## Data Availability Statement

Datasets presented in this article are available at the European Nucleotide Archive (ENA) under the project ID: PRJEB38585.

## Author Contributions

CK, RT, and FR-T contributed to the design and implementation of the research, to the analysis of the results, and to the writing of the manuscript. All authors contributed to the article and approved the submitted version.

## Conflict of Interest

The authors declare that the research was conducted in the absence of any commercial or financial relationships that could be construed as a potential conflict of interest.

## Footnotes

1 http://repeatmasker.org

2 http://mafft.cbrc.jp/alignment/software/

3 http://bioinformatics.ibp.cz:9999/#/en/palindrome

4 https://tandem.bu.edu/trf/trf.html

5 https://nonb-abcc.ncifcrf.gov/apps/nBMST/default

6 https://www.fruitfly.org/seq_tools/promoter.html

7 http://floresta.eead.csic.es/footprintdb

8 http://redfly.ccr.buffalo.edu/

9 http://dsubobscura.serveftp.com/
