# Supplementary material for: The Cyclically Seasonal Drosophila subobscura Inversion O7 Originated From Fragile Genomic Sites and Relocated Immunity and Metabolic Genes
Source: Front Genet. 2020 Oct 9;11:565836. doi: 10.3389/fgene.2020.565836 (PMC7584159; doi:10.3389/fgene.2020.565836)
Supplement: Supplementary file 3 [file Data_Sheet_1.PDF]

Supplementary Material

Supplementary Figure S1. Isolation of segments A and B for reconstruction of the proximal breakpoint of O_7. **(A)** MSA of the [+A|+B] region from the uninverted state (Dg, Ds_ch-cu, and Ds_B) with the [+A|-C] region from O_7. The regions of O_7 corresponding to segments A and C are denoted with capital and lower-case letters, respectively. **(B)** MSA of the [+A|+B] region from the uninverted state (Dg, Ds_ch-cu, and Ds_B) with the reverse complement (RC) of the [−B|+D] region from O_7. The regions of O_7 corresponding to segments D and B are denoted with lower-case and capital letters, respectively. Black and grey backgrounds denote invariant and 75% conserved MSA columns, respectively. Numbers in brackets are basepair distances to the nearest coding sequence.

Supplementary Figure S2. Isolation of segments C and D for reconstruction of the distal breakpoint of O_7. **(A)** MSA of the [+C|+D] region from the uninverted state (Dg, Ds_ch-cu, and Ds_B) with the reverse complement (RC) of the [+A|-C] region from O_7. The regions of O_7 corresponding to segments C and A are denoted with capital and lower-case letters, respectively. **(B)** MSA of the [+C|+D] region from the uninverted state (Dg, Ds_ch-cu, and Ds_B) with the reverse complement (RC) of the [−B|+D] region from O_7. The regions of O_7 corresponding to segments B and D are denoted with lower-case and capital letters, respectively. Black and grey backgrounds denote invariant and 75% conserved MSA columns, respectively. Numbers in brackets are basepair distances to the nearest coding sequence.

Supplementary Figure S3. MSA of the proximal breakpoint region of O_7, including the [+A|+B] region from the uninverted state (Dg, Ds_ch-cu, and Ds_B) and the [+A|+B] region of O_7 reconstructed by concatenation of the identified segments A and B. The breakpoint junction between segments A and B is located between the two corresponding yellow boxes above the aligned sequences. Colored boxes below the aligned sequences denote: sepia, a 153bp-long direct repeat; purple, copies A and B of duplicate d1; and red, copies A and B of duplicate d2. The copies of d1 and d2 are oriented as predicted from the proposed scenario for the origin of O_7 (see also Figure 3). Also indicated are the proximal and distal ends of the microinversion, and the proximal end of O_7 Black and grey backgrounds denote invariant and 75% conserved MSA columns, respectively. Numbers in brackets are basepair distances to the nearest coding sequence.

Supplementary Figure S4. The proximal breakpoint after undoing the microinversion. Similar to Supplementary Figure S3, but with the microinversion reversed.

Supplementary Figure S5. Ancestral form of the proximal breakpoint before the ocurrance of the DSBs. Similar to Supplementary Figure S4, but with one copy of each of d1 and d2 eliminated.

Supplementary Figure S6:
MSA of the distal breakpoint region of O_7, including the [+C|+D] region from the uninverted state (Dg, Ds_ch-cu, and Ds_B) and the [+C|+D] region of O_7 reconstructed by concatenation of the identified segments C and D. The breakpoint junction between segments C and D is located between the two corresponding yellow boxes above the aligned sequences. Colored boxes above the aligned sequences denote: sepia, the two IRs; black and pink within the IRs, two exons and one intron of each of *AttA2a* and *AttA2b*; light blue, the regions of the central spacer of O_7 between the two IRs that are similar to either Ds_ch-cu or Ds_B and Dg; dar blue within the central spacer, the CSR; red, putative repair-associated filler DNA (see also Figure 3). Black and grey backgrounds denote invariant and 75% conserved MSA columns, respectively. Numbers in brackets are basepair distances to the nearest coding sequence.

Supplementary Figure S7. Maximum likelihood tree of *Drosophila Attacin* genes. The tree includes 63 homologous nucleotide coding sequences (see Supplementary Table 1) with 250 codon sites. Numbers indicate bootstrap support values of IQ-Tree analysis (1000 replicates) with the MGK+F1X4+G4 model. The tree was rooted at the midpoint between the most divergent *Attacins*. The scale bar denotes the estimated number of nucleotide substitutions per site.

Supplementary Table 1. Synteny relationships for *Attacin* genes across *Drosophila*. The MS Excel file contains six spreadsheets, including one for this title, and one for each of five major *Drosophila Attacin* family members (i.e., A-B, A2, A3, C and D). For each *Attacin*, columns "A" to "C" list the taxonomy of the sequences, including the subgenus within *Drosophila*, the group within the *Drosophila* subgenus, and the species. Columns "D" and "E" list the Muller element and the corresponding chromosome of the *Attacin* location. The remaining columns list the Attacin genes with the three upstream and downstream flanking genes. *Attacin* genes are highlighted in red, and syntenic orthologous flanking genes in yellow.

A

Identification of segment A of the proximal breakpoint

MSA of [+A|+B] from the uninverted state (Dg, Ds_ch-cu, and Ds_B) with [+A|−C] from the inverted state (Ds_7)

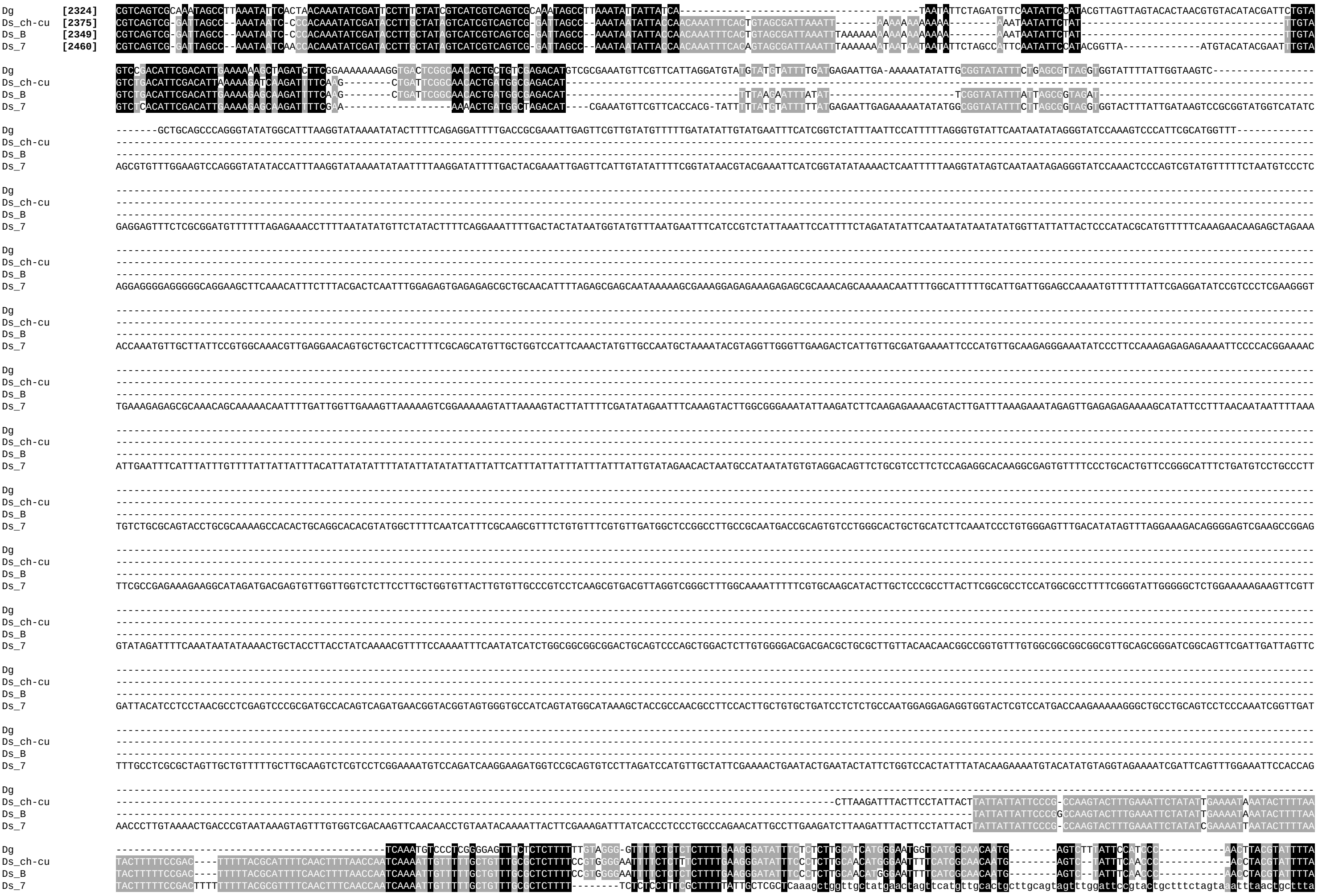

B

Identification of segment B of the proximal breakpoint

MSA of [+A|+B] from the uninverted state (Dg, Ds_ch-cu, and Ds_B) with RC[−B|+D] from the inverted state (Ds_7)

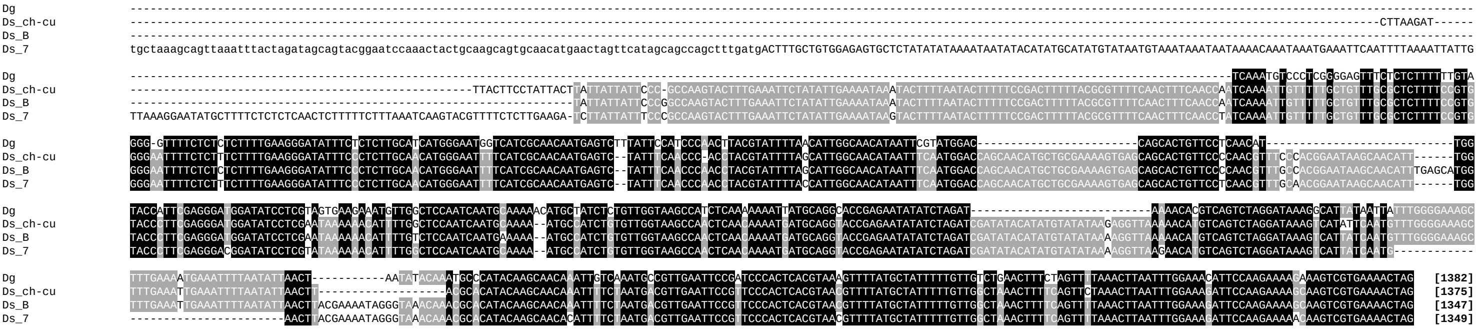

Supplementary Figure S1

A

Identification of segment C of the distal breakpoint

MSA of [+C|+D] from the uninverted state (Dg, Ds_ch-cu, and Ds_B) with RC[+A|−C] from the inverted state (Ds_7)

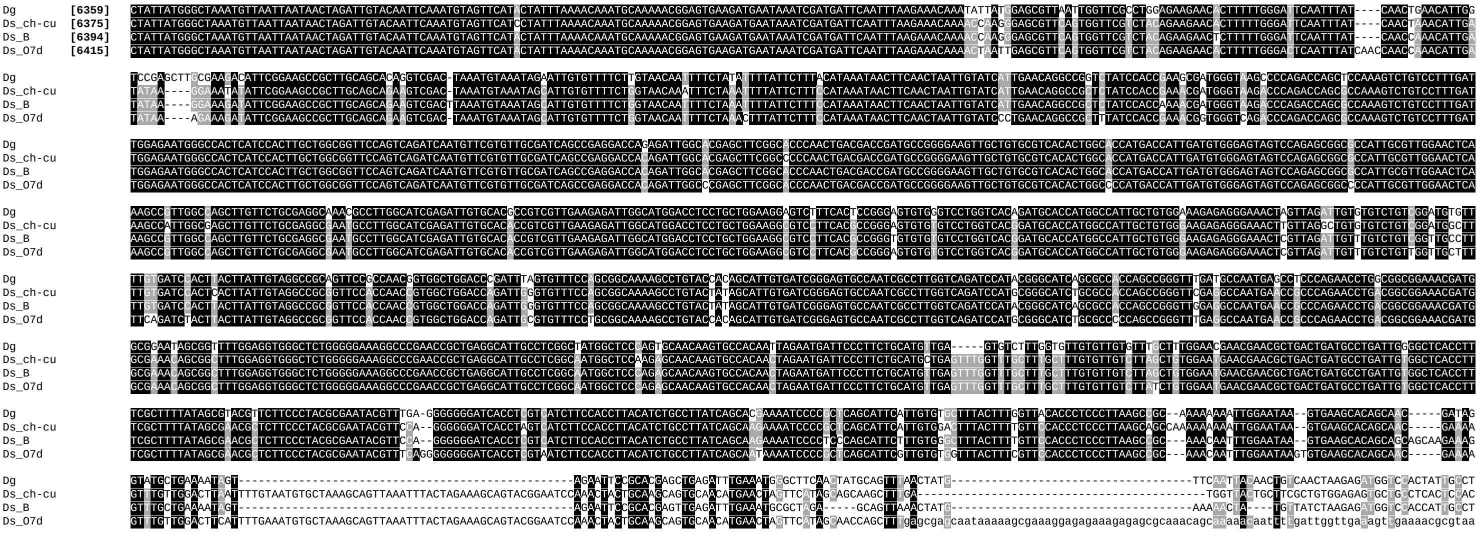

B

Identification of segment D of the distal breakpoint

MSA of [+C|+D] from the uninverted state (Dg, Ds_ch-cu, and Ds_B) with [−B|+D] from the inverted state (Ds_7)

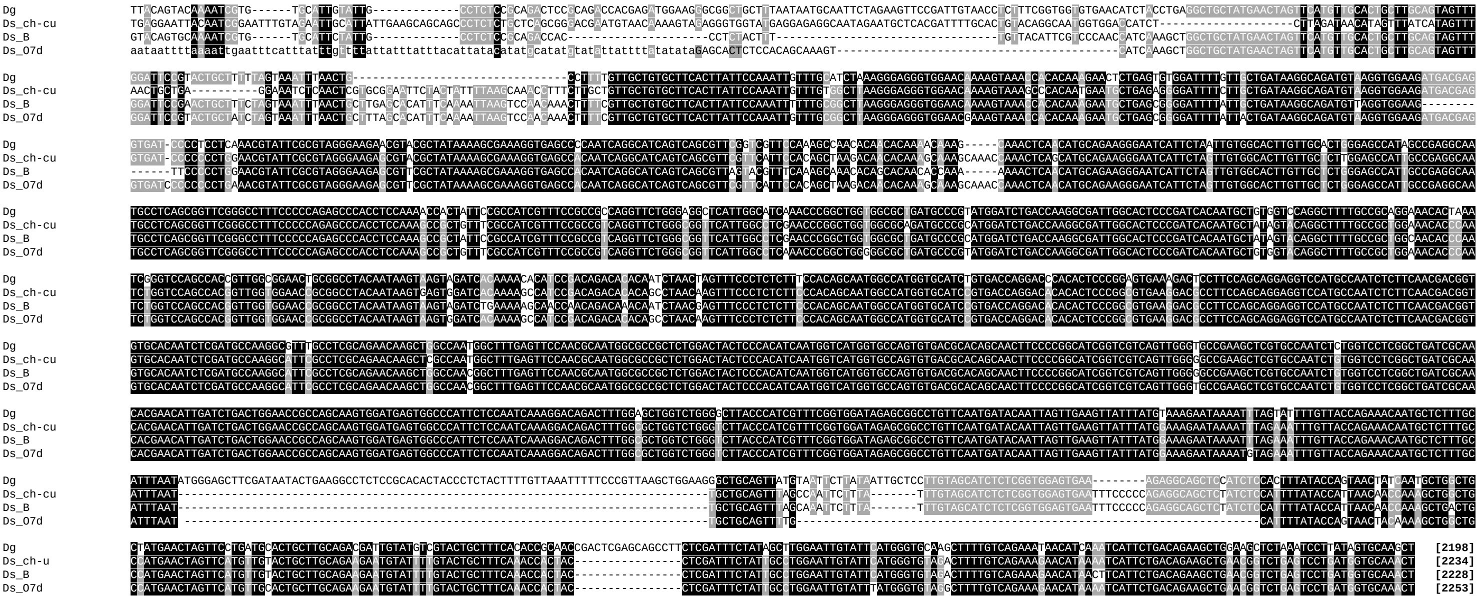

Supplementary Figure S2

Proximal breakpoint [+A|+B]

MSA of [+A|+B] from the uninverted state (Dg, Ds_ch-cu, and Ds_B) with reconstructed [+A|+B] from the inverted state (Ds_7)

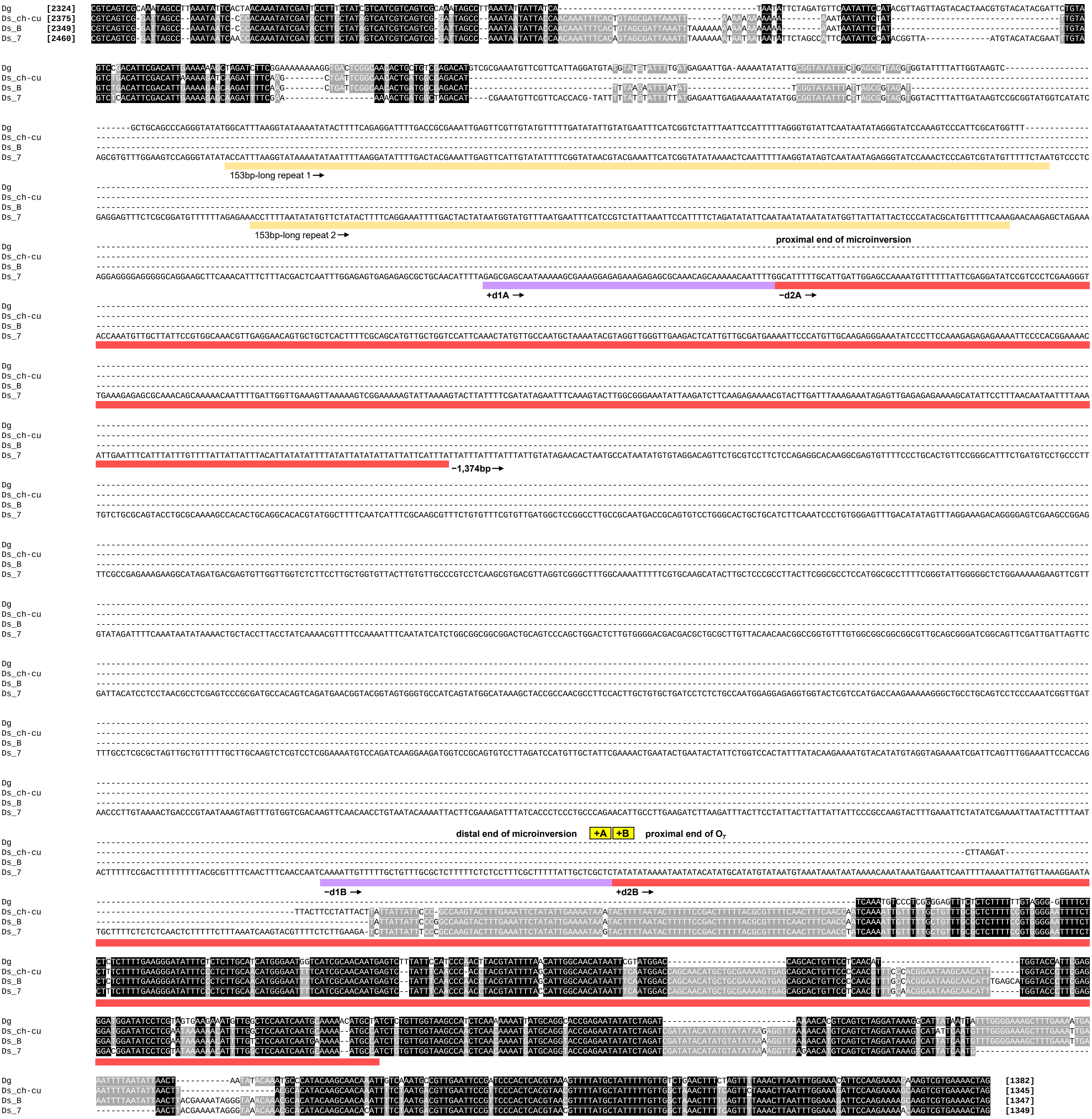

Supplementary Figure S3

Proximal breakpoint [+A|+B] with the microinversion reversed

MSA of [+A|+B] from the uninverted state (Dg, Ds_ch-cu, and Ds_B) with reconstructed [+A|+B] from the inverted state (Ds_7)

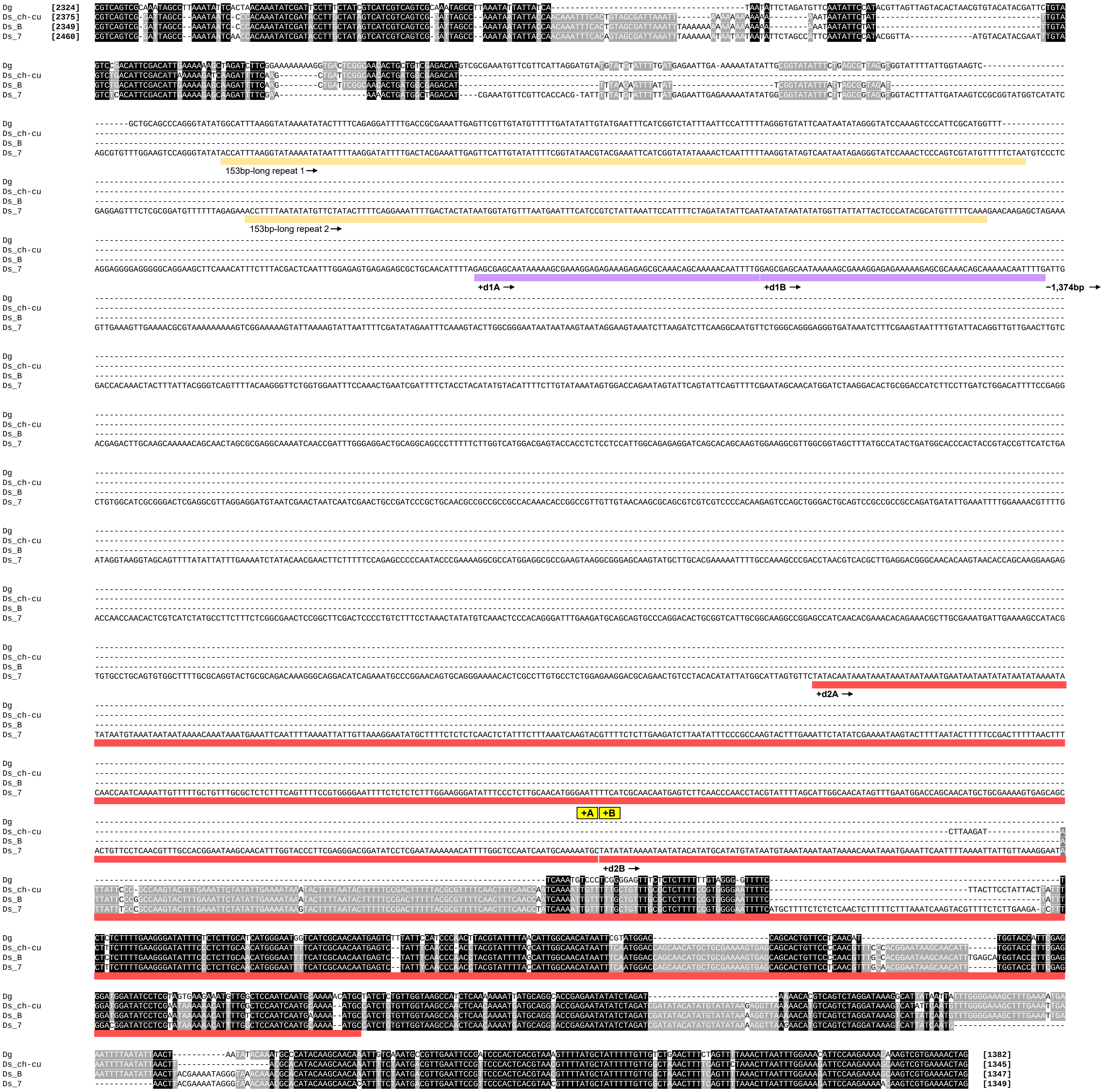

Supplementary Figure S4

Proximal breakpoint [+A|+B] as before the origination of O_7

MSA of [+A|+B] from the uninverted state (Dg, Ds_ch-cu, and Ds_B) with reconstructed [+A|+B] from the inverted state (Ds_7) as it was before O_7 arose

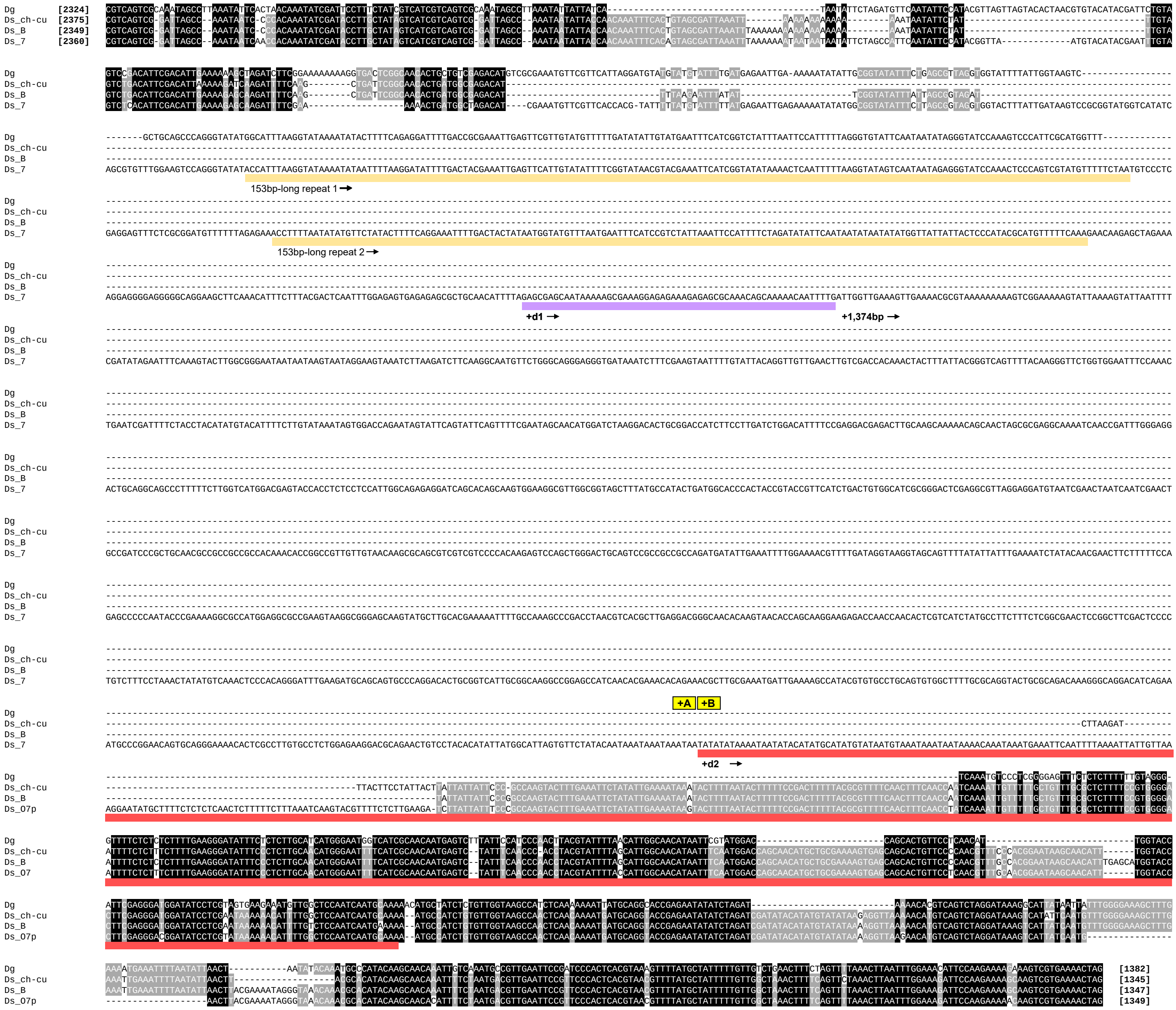

Supplementary Figure S5

Distal breakpoint [+C|+D]

MSA of [+C|+D] from the uninverted state (Dg, Ds_ch-cu, and Ds_B) with reconstructed [+C|+D] from the inverted state (Ds_7)

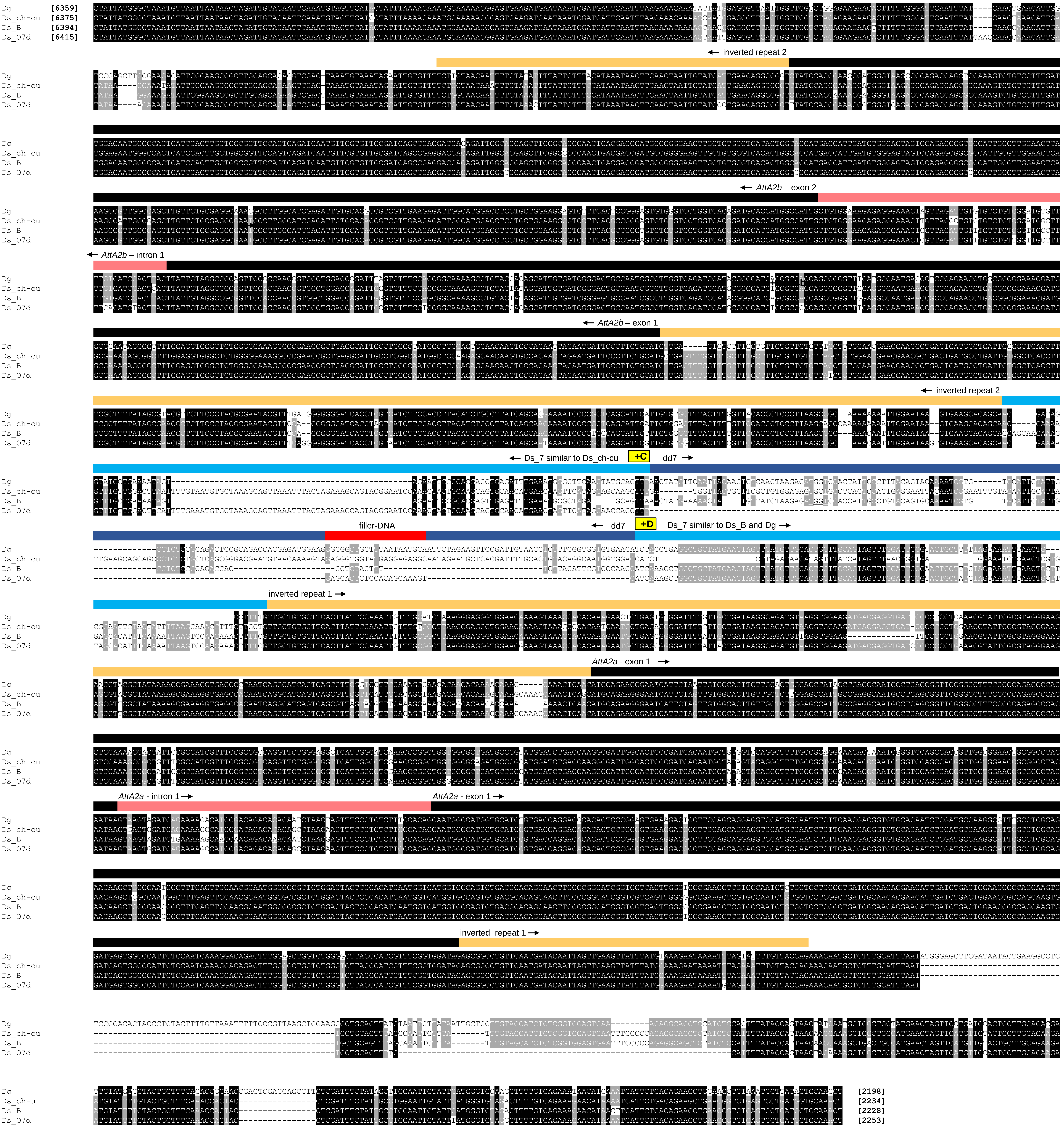

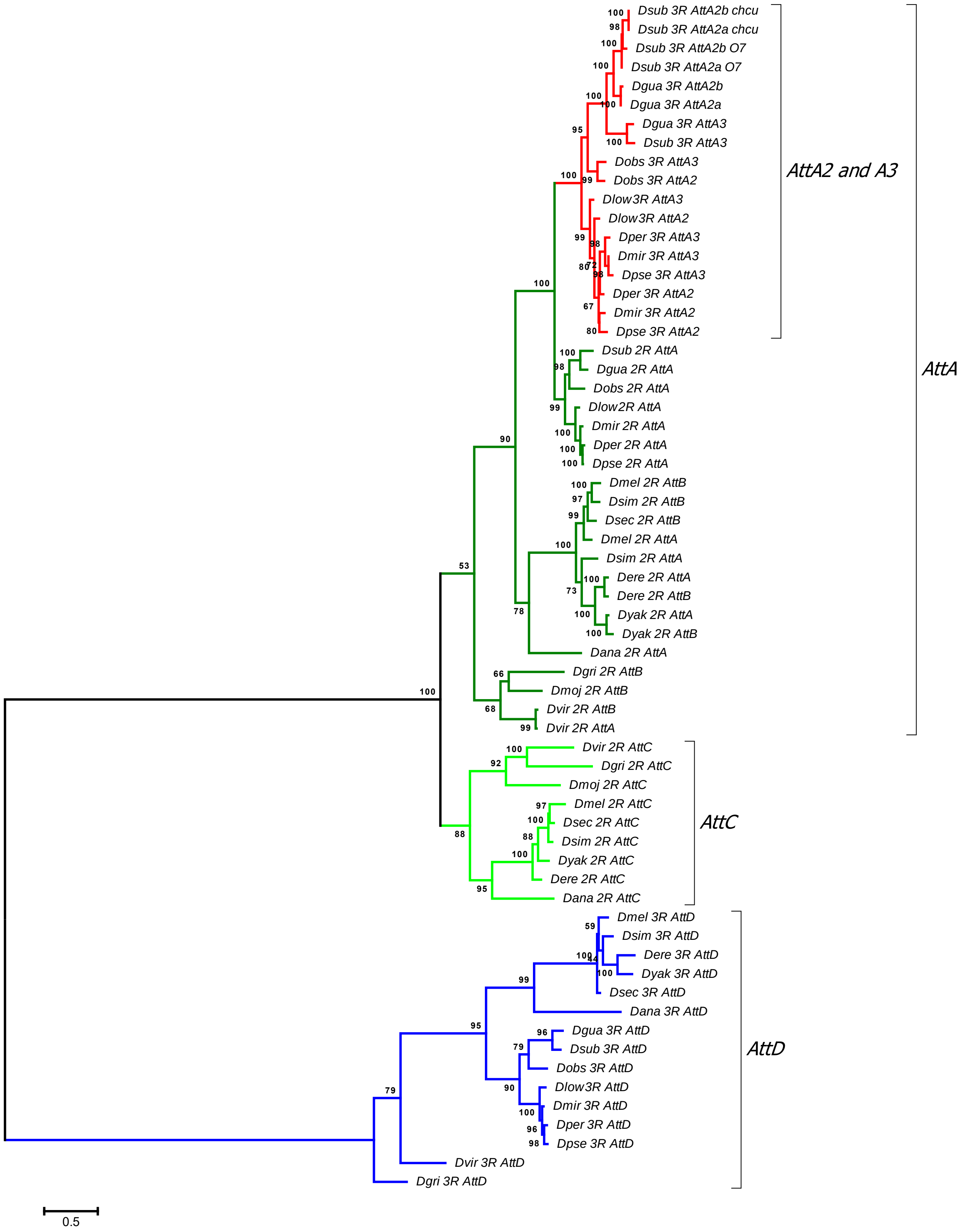

Supplementary Figure S7
